# Impact of somatic *PIK3CA* mutations on clinical outcomes in HER2-positive breast cancer

**DOI:** 10.1186/s13058-026-02324-6

**Published:** 2026-06-20

**Authors:** Nickolas Stabellini, Sonia D. de Oliveira, Takae Mizukami, Shawn M. Sweeney, Nikolaus Schultz, Ken Kehl, Gregory Riely, Katherine Panageas, Deborah Schrag, Philippe Bedard, Christine Micheel, Xindi Guo, Alberto J. Montero

**Affiliations:** 1 Case Western Reserve University, Cleveland, OH USA; 2 Department of Hematology-Oncology, University Hospitals Seidman Cancer Center, Cleveland, OH USA; 3 Immunomonitoring Laboratory, Department of Cancer Sciences, Cleveland Clinic, Cleveland, OH USA; 4 Division of Cardiology, Department of Medicine, Medical College of Georgia at Augusta University, Augusta, GA USA; 5 American Association for Cancer Research (AACR), Philadelphia, PA USA; 6 Memorial Sloan Kettering Cancer Center (MSKCC), New York, NY USA; 7 Dana-Farber Cancer Institute (DFCI), Boston, MA USA; 8 Princess Margaret Cancer Centre, University Health Network (UHN), Toronto, ON Canada; 9 Vanderbilt University Medical Center (VUMC), Nashville, TN USA; 10 Sage Bionetworks, Seattle, WA USA

**Keywords:** *PIK3CA*, Mutation, Survival, Progression, Recurrence, Breast cancer, HER2-positive

## Abstract

**Background:**

HER2-positive (HER2+) breast cancer (BC) accounts for 15–20% of all BC. Mutations in the *PIK3CA* gene are found in 25–30% of HER2 + BC cases and have been implicated in tumor progression and endocrine therapy resistance. Recent evidence suggests that a somatic *PIK3CA* mutation (*PIK3CA*m) may serve as a marker of early progression in HER2 + metastatic BC. However, its precise prognostic significance across various lines of therapy and disease stages is not fully understood. We therefore aimed to evaluate the impact of *PIK3CA*m status on clinical outcomes in HER2 + BC patients.

**Methods:**

We conducted a retrospective cohort study using data from the American Association for Cancer Research (AACR) Project GENIE Biopharma Collaborative (BPC). Overall survival (OS), Distant Recurrence-Free Survival (DRFS), and progression-free survival (PFS) were the primary outcomes. We compared patients with and without *PIK3CA*m and performed survival analysis using Kaplan-Meier methods and Cox proportional hazards models.

**Results:**

We identified 212 HER2 + patients (150 early-stage and 62 Stage IV), of which 58 had a *PIK3CA*m. The median OS for the overall cohort was 125.1 months from treatment. Among early-stage patients, *PIK3CA*m was associated with a longer DRFS from diagnosis (37.7 months [95% CI 28.6–62.6] vs. 22 months [95% CI 16.8–32.8], *p* = 0.04). In contrast, among patients with Stage IV disease receiving first-line therapy, *PIK3CA*m was associated with a shorter median PFS (5.5 [95% CI 1.6–32.2] vs. 14.9 months [95% CI 7.2–22.0], *p* = 0.04). *PIK3CA*m was not identified as an independent predictor of OS, DRFS, or PFS in univariable or multivariable regression analyses. Within the *PIK3CA*m subgroup, not having received hormone therapy was the only factor associated with worse survival on multivariable analysis (aHR = 4.51, 95% CI 1.77–11.5).

**Conclusion:**

*PIK3CA*m was not found to be associated with worse OS, however it was associated with a shorter PFS among patients receiving first-line therapy, suggesting *PIK3CA*m could be explored as a predictive biomarker for identifying HER2 + Stage IV patients at higher risk of disease progression. These findings underscore the need for enhanced surveillance and personalized treatment strategies to manage disease progression in this patient subgroup.

**Supplementary Information:**

The online version contains supplementary material available at https://doi.org/10.1186/s13058-026-02324-6.

## Introduction

Female breast cancer (BC) is the most diagnosed malignancy in the world [1]. Worldwide, in 2022, it was responsible for 2,308,897 cases (11.6% of all cancer cases) and 665,684 related deaths (6.9% of all cancer-related deaths) [1]. In the United States (U.S.), BC was estimated to be responsible for 319,750 new cases and 42,680 related deaths in 2025 [2].

Newly diagnosed BC cases are routinely tested for estrogen (ER) and progesterone (PR) receptor expression, as well as for overexpression of human epidermal growth factor receptor 2 (HER2), which together guide both prognostic evaluation and therapeutic decision-making [3]. Among these, HER2-positive (HER2+) BC accounts for approximately 15–20% of all diagnoses, identifying patients who benefit from HER2-targeted treatments [3, 4]. Historically, HER2 + BC was considered an aggressive subtype, given the greater metastatic potential, but the development of HER2-targeted therapies has dramatically improved survival outcomes [5]. Currently, patients with early-stage HER2 + BC treated with chemotherapy and dual antibody therapy can achieve 3-year invasive-disease-free survival rates above 90%, and more than half of patients with metastatic HER2 + disease are diagnosed *de novo* BC [5–7].

The phosphatidylinositol-3-kinase (PI3K) pathway, driven by the *PIK3CA* gene, plays a critical role in tumor initiation, growth, proliferation, and therapy resistance [8]. After the TP53 tumor suppressor gene, the *PIK3CA* oncogene is the most frequently mutated gene in BC [9–11]. *PIK3CA* mutations (*PIK3CA*m) are estimated to occur in about 35% of all BC cases, most commonly in ER-positive and HER2-negative subtypes ] [12]. In HER2 + BC, these mutations are found in approximately 25–30% of cases [13]. *PIK3CA*m has been reported to be associated with shorter survival times in metastatic breast cancer (mBC), reduced effectiveness of HER2-targeted therapies, and resistance to endocrine therapy [14, 15]. In the recently presented phase 3 Destiny Breast09 trial, the presence of a *PIK3CA*m was identified as a marker of early progression in HER2 + metastatic BC (mBC) receiving first-line therapy with trastuzumab/pertuzumab monoclonal antibodies [16].

Although *PIK3CA*m is consistently associated with lower rates of pathological complete response (pCR) in the neoadjuvant setting for HER2 + patients and with a poorer prognosis, its precise prognostic significance across various lines of therapy and disease stages in HER2 + BC is not fully understood. Therefore, we aimed to evaluate the impact of *PIK3CA*m status on clinical outcomes in HER2 + BC patients.

## Methods

### Data source

Data was obtained from the American Association for Cancer Research (AACR) Project Genomics Evidence Neoplasia Information Exchange Biopharma Collaborative (GENIE BPC) Breast Cancer Cohort version 1.2 [17–19]. The GENIE BPC is a multi-phase, pre-competitive collaboration between 10 biopharmaceutical companies and select GENIE-participating academic institutions [17]. It focuses on providing detailed clinical annotations for a subset of patients within the GENIE Registry [17].

The data available integrates demographic, diagnosis, genomic, and treatment information with longitudinal, real-world patient outcomes [17]. Data collection follows a structured framework to ensure interoperability and forward compatibility with other data models [17]. Each cohort undergoes a series of rigorous quality control and assurance protocols, which ensures consistency, accuracy, and reliability of the data across multiple institutions before its public release [17].

The biopharma collaborative datasets consist of patient- and sample-level data sourced from the AACR Project GENIE Registry [17]. This is supplemented with additional longitudinal clinical data, which is collected from institutional electronic health records and tumor registries into local REDCap databases [17]. Curators utilize the PRISSMM™ framework for this collection [17, 20, 21]. PRISSMM™ standardizes data collection from Pathology, Radiology, Imaging, Signs and Symptoms, tumor Markers, and Medical oncologist assessments, combining these data with patient demographics and treatments [17, 20, 21].

### Study design

**Fig. 1:**
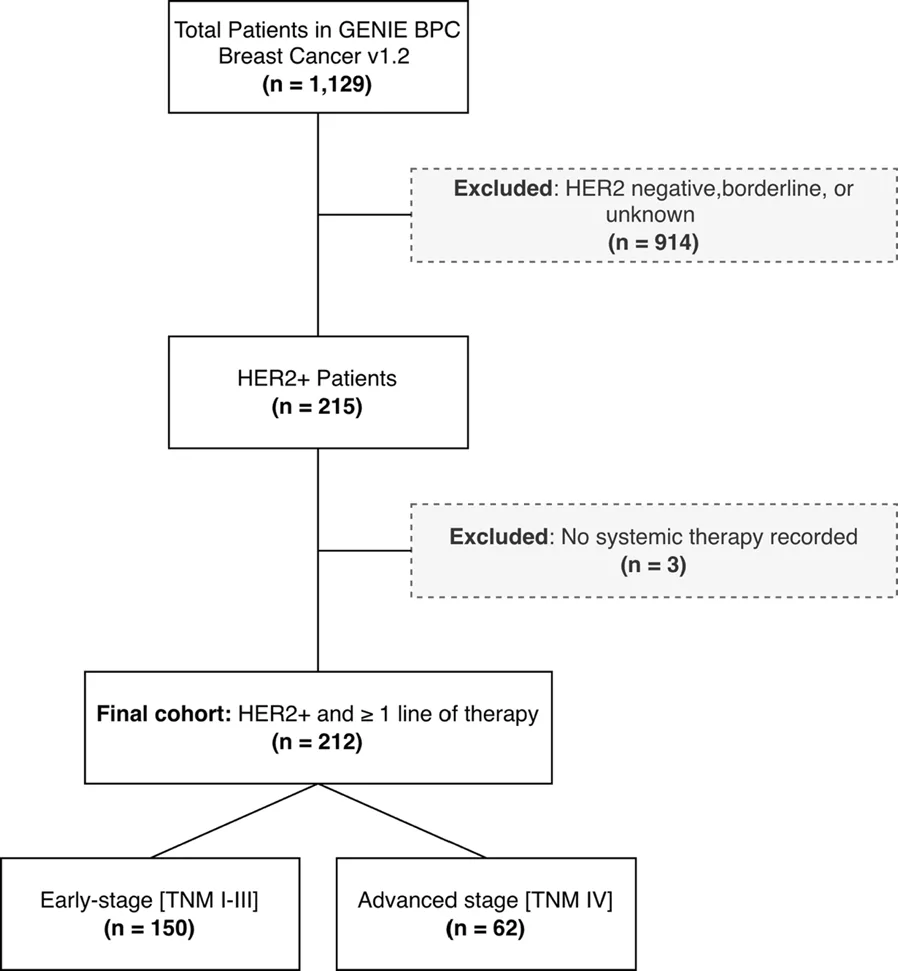
CONSORT diagram of study cohort selection

The study was designed as a retrospective cohort. The BC cohort of the GENIE BPC includes patients diagnosed between 18 and 56 years of age who underwent tumor sequencing between 2013 and 2020. Of those, our study included HER2 + patients who had received at least one line of therapy (Fig. 1).

### Outcomes

The primary outcomes were overall survival (OS), Distant Recurrence-Free Survival (DRFS; calculated for early-stage patients), and progression-free survival (PFS; calculated for those with Stage IV disease).

### Covariates

Patient and clinical characteristics, treatment history, and genomic alterations were included (Supplemental Table 1).

Demographics included age at diagnosis, sex, race, and ethnicity. Tumor Characteristics encompassed cancer sequence, tumor laterality, stage at diagnosis, BC histology, ER status, PR status, HER2 status, and BC subtype. Treatment-Related Factors comprised neoadjuvant therapy before pathologic staging, number of regimens, details of the first regimen (e.g., number of drugs, clinical trial participation, duration), and the receipt of specific therapy types (chemotherapy, hormone therapy, targeted/immunotherapy; defined by drug names according to Supplemental Table 2). Distant Metastasis included the presence and site of distant metastasis at diagnosis. Clinical Procedures and Markers involved the number of imaging reports, types of scans received (CT, MRI, PET, Bone Scan, Mammogram), number of pathology reports, number of medical oncologist visits, and tumor marker results (CA 15 − 3, CA 27.29). Genomic Alterations included the alteration status of key genes (e.g., *TP53*,* PIK3CA*,* ERBB2*) and pathways (e.g., p53, PI3K, cell cycle, RTK/RAS). Sequencing Data provided details about the cancer panel tests (e.g., year of sequencing, time from diagnosis to report, specimen histological subtype).

### Statistical analysis

Descriptive analysis summarized continuous variables as mean and standard deviation (SD) if normally distributed, or as median and interquartile range (IQR; 25th and 75th percentiles) if non-normally distributed. Data distribution was assessed using the Shapiro-Wilk test and histograms. Categorical variables were summarized as frequencies and percentages.

Comparative statistics, comparing patients with *PIK3CA*m versus those without, were performed using Fisher’s exact test for categorical variables. For continuous covariates, independent samples t-tests were used for normally distributed variables, and the non-parametric Kruskal-Wallis test was used for non-normally distributed variables.

Survival analyses were performed using Kaplan-Meier to estimate OS, DRFS, and PFS. The log-rank test was used to compare survival distributions overall and across subgroups, including *PIK3CA* mutation status, ER status (positive vs. negative), and TNM stage (early-stage [TNM I–III] vs. Stage IV), with TNM analyses also performed stratified by line of therapy (first- vs. second-line).

Univariable Cox proportional hazards (COX PH) models were used to assess the association between individual covariates and outcomes, and results were reported as hazard ratios (HR), 95% confidence intervals (CI), and p-values. Multivariable COX PH were constructed using a LASSO (Least Absolute Shrinkage and Selection Operator) regression approach for variable selection. Variables were first filtered based on a minimum alteration frequency (for genomic data) and then for near-zero variance. Highly correlated variables (Pearson correlation coefficient > 0.70) were also identified and handled during the LASSO process to ensure model stability. A 10-fold cross-validation was performed to identify the optimal penalty parameter (λ). The λ min (lambda.min) value, which corresponds to the λ that gives the minimum mean cross-validated error, was chosen for selecting the final set of covariates. The selected variables from the LASSO model were then included in the final multivariable COX PH models. All models were performed after checking if assumptions were met. Cox models for OS were performed in the overall cohort and stratified by ER status, TNM stage, and line of therapy. For DRFS and PFS, stratified analyses by ER status and line of therapy were not performed. No interaction analyses were conducted.

All statistical analyses were performed using R (version 4.5.0) [22]. A* p*-value of < 0.05 was considered statistically significant.

## Results

We included 212 HER2 + patients (Table 1; Supplemental Tables 3–6), with a median age at diagnosis of 43 years (IQR 37–48). Most were female (99.1%), White (77%) and non-Hispanic (95%), and presented with Stage II disease (12%). 98% of patients received cytotoxic chemotherapy, 92% received HER2 therapy, 67% endocrine therapy, 14% CDK4/6 inhibitors, 9% PIK3/mTOR inhibitors, and 9% received immunotherapy.

**Table 1:** Overall patient characteristics of HER2 + patient cohort and by *PIK3CA* Mutation status

Characteristic	Overall	*PIK3CA* not altered	*PIK3CA* altered	*p*-value^2^
	*N* = 212^1^	*N* = 154^1^	*N* = 58^1^	
Age at diagnosis	43 (37, 48)	42 (37, 48)	43 (37, 46)	0.8
Race	–	–	–	0.8
White	163 (77%)	117 (76%)	46 (79%)	–
Black	16 (7.5%)	12 (7.8%)	4 (6.9%)	–
Other	12 (5.7%)	8 (5.2%)	4 (6.9%)	–
Unknown/Other	21 (9.9%)	17 (11%)	4 (6.9%)	–
Ethnicity	–	–	–	0.9
Hispanic	7 (3.3%)	6 (3.9%)	1 (1.7%)	–
Non-Hispanic	201 (95%)	145 (94%)	56 (97%)	–
Unknown	4 (1.9%)	3 (1.9%)	1 (1.7%)	–
Stage at diagnosis	–	–	–	0.5
Stage I-III	150 (71%)	107 (69%)	43 (74%)	–
Stage IV	62 (29%)	47 (31%)	15 (26%)	–
Histology	–	–	–	0.7
Invasive ductal carcinoma	204 (96%)	148 (96%)	56 (97%)	–
Invasive lobular carcinoma	6 (2.8%)	5 (3.2%)	1 (1.7%)	–
Other histology	2 (0.9%)	1 (0.6%)	1 (1.7%)	–
ER status	–	–	–	**0.007**
Negative	80 (38%)	67 (44%)	13 (22%)	–
Positive	132 (62%)	87 (56%)	45 (78%)	–
PR status	–	–	–	0.06
Negative	90 (42%)	72 (47%)	18 (31%)	–
Positive	121 (57%)	81 (53%)	40 (69%)	–
Unknown/Not tested	1 (0.5%)	1 (0.6%)	0 (0%)	–
***PIK3CA*** mutation type	–	–	–	**< 0.001**
Kinase domain (H1047)	24 (11%)	0 (0%)	24 (41%)	–
Helical domain (E545)	7 (3.3%)	0 (0%)	7 (12%)	–
Other *PIK3CA* mutation	26 (12%)	0 (0%)	26 (45%)	–
Received chemotherapy	207 (98%)	152 (99%)	55 (95%)	0.13
Received hormone therapy	142 (67%)	97 (63%)	45 (78%)	**0.04**
Received immunotherapy	19 (9.0%)	14 (9.1%)	5 (8.6%)	> 0.9
Received HER2 therapy	194 (92%)	145 (94%)	49 (84%)	**0.04**
Received CDK4/6 inhibitors	29 (14%)	17 (11%)	12 (21%)	0.07
Received PI3K/mTOR inhibitors	19 (9.0%)	11 (7.1%)	8 (14%)	0.2

^1^Median (Q1, Q3); n (%)

The bold *p*-values indicate statistical significance (*p* < 0.05)

Of the 212 patients included in our study, 58 had a *PIK3CA*m, of which 24 (41%) were kinase domain mutations (Table 1). Comparing those with *PIK3CA*m versus those without (Table 1; Supplemental Tables 3–6), patients with *PIK3CA*m had higher rates of ER-positive disease (78% vs. 56%, *p* = 0.007), and higher rates of receiving endocrine therapy (78% vs. 63%, *p* = 0.04). Conversely, the presence of a *PIK3CA*m was associated with lower rates of HER2 therapy (84% vs. 94%, *p* = 0.04). The time from initial diagnosis to distant metastasis in early-stage patients was significantly longer for those with a *PIK3CA*m (median: 38 months [IQR: 23–73] vs. 22 months [IQR: 10–43], *p* = 0.01; Supplemental Table 3). Furthermore, as expected, patients with *PIK3CA*m had higher rates of altered PI3K pathway (100% vs. 21%, *p* < 0.001). Interestingly, they also had significantly lower rates of Hippo pathway alteration (0% vs. 11%, *p* = 0.01).

### Impact of * PIK3CA*m on survival outcomes

The median OS from initial BC diagnosis was 124.4 months (95% CI 98.4-157.2; Table 2). In univariable COX PH analysis, *PIK3CA*m was not significantly associated with worse OS outcomes (HR = 1.06, 95% CI 0.68–1.66; Table 3). LASSO did not select *PIK3CA*m for the set of covariates in multivariable COX PH analysis (Table 3). Similar results were found in subgroup analyses by stage and by ER-status (Supplemental Tables 7–10).

**Table 2:** Survival Summary by HER2 + subgroup and endpoint

					Median Survival			2-Year Survival			5-Year Survival			10-Year Survival		
	Status	*N*	Events	*p*-value	Median survival (Months)	95% CI lower	95% CI upper	2-Year prob.	95% CI lower	95% CI upper	5-year prob.	95% CI lower	95% CI upper	10-year prob.	95% CI lower	95% CI upper
*HER2+*																
OS from diagnosis	*PIK3CA* altered	58	29	0.807	126	98.8	NA	96.50%	0.918	1	69.90%	0.586	0.835	51.90%	0.39	0.691
	*PIK3CA* not altered	154	56		120.9	87.9	157.2	97.30%	0.947	0.999	81.40%	0.746	0.888	51.40%	0.413	0.641
	Overall	212	85	-	124.4	98.4	157.2	97.10%	0.948	0.994	77.90%	0.719	0.844	51.60%	0.433	0.615
OS from treatment	*PIK3CA* altered	57	28	0.666	125.1	98.3	NA	96.40%	0.917	1	71.50%	0.602	0.849	52.20%	0.39	0.7
	*PIK3CA* Not altered	152	55		131.2	87.7	157.7	95.90%	0.927	0.992	78.50%	0.712	0.866	54.80%	0.448	0.67
	Overall	209	83	-	125.1	98.3	155.3	96.00%	0.934	0.988	76.50%	0.703	0.833	53.80%	0.455	0.637
*HER2 + ER negative*																
OS from diagnosis	*PIK3CA* altered	13	8	0.236	44.9	37.6	NA	100.00%	1	1	36.70%	0.168	0.798	18.30%	0.037	0.898
	*PIK3CA* Not altered	67	29		86.4	65	NA	93.80%	0.88	0.999	70.90%	0.594	0.845	35.80%	0.216	0.593
	Overall	80	37	-	85.8	64.2	140.1	94.80%	0.899	0.999	64.80%	0.539	0.78	32.80%	0.202	0.533
OS from treatment	*PIK3CA* altered	12	7	0.247	44	34.8	NA	100.00%	1	1	40.40%	0.19	0.859	20.20%	0.042	0.979
	*PIK3CA* not altered	66	28		85.2	66.1	NA	90.40%	0.834	0.98	71.80%	0.603	0.855	45.40%	0.312	0.66
	overall	78	35	-	85.2	65.4	142	91.90%	0.858	0.983	66.90%	0.559	0.799	41.30%	0.283	0.603
*HER2 + ER positive*																
OS from diagnosis	*PIK3CA* altered	45	21	0.597	152.9	103.3	NA	95.60%	0.897	1	78.70%	0.672	0.922	59.80%	0.455	0.785
	*PIK3CA* not altered	87	27		153.1	98.4	NA	100.00%	1	1	89.30%	0.819	0.973	62.50%	0.499	0.782
	Overall	132	48	-	153.1	120.9	168.9	98.50%	0.964	1	85.30%	0.788	0.923	61.10%	0.511	0.729
OS from treatment	*PIK3CA* altered	45	21	0.565	151.3	102.1	NA	95.60%	0.897	1	78.70%	0.672	0.922	58.90%	0.444	0.782
	*PIK3CA* not altered	86	27		151.3	97.4	NA	100.00%	1	1	83.50%	0.745	0.936	61.80%	0.491	0.779
	Overall	131	48	-	151.3	120.3	167.1	98.40%	0.963	1	81.90%	0.747	0.897	60.40%	0.504	0.725
*HER2 + Early stage (I-III)*																
OS from diagnosis	*PIK3CA* altered	43	22	0.907	152.9	98.8	NA	100.00%	1	1	74.90%	0.625	0.897	55.50%	0.41	0.751
	*PIK3CA* not altered	107	41		134.8	86.4	168.4	96.10%	0.925	0.999	78.80%	0.704	0.883	53.70%	0.424	0.679
	Overall	150	63	-	134.8	98.4	163	97.30%	0.947	0.999	77.60%	0.705	0.854	54.10%	0.449	0.653
OS from treatment	*PIK3CA* altered	42	21	0.955	151.3	98.3	NA	100.00%	1	1	77.10%	0.65	0.915	56.10%	0.412	0.764
	*PIK3CA* not altered	106	41		131.2	87.3	162.8	96.10%	0.924	0.999	73.80%	0.645	0.845	55.00%	0.437	0.692
	Overall	148	62	-	131.2	98.3	160.6	97.20%	0.946	0.999	75.10%	0.676	0.834	55.20%	0.458	0.665
DRFS from diagnosis	*PIK3CA* altered	34	34	***0.048***	37.7	28.6	62.6	73.50%	0.601	0.9	35.30%	0.224	0.556	2.90%	0.004	0.203
	*PIK3CA* not altered	81	81		22	16.8	32.8	48.10%	0.384	0.604	18.50%	0.117	0.292	3.70%	0.012	0.112
	Overall	115	115	-	26.3	21.8	35.5	55.70%	0.473	0.655	23.50%	0.169	0.327	3.50%	0.013	0.091
DRFS from treatment	*PIK3CA* altered	32	32	0.111	36.9	27.7	61.7	71.90%	0.579	0.893	34.40%	0.213	0.555	3.10%	0.005	0.215
	*PIK3CA* not altered	72	72		22.4	19.2	33.5	45.80%	0.357	0.589	19.40%	0.122	0.311	4.20%	0.014	0.126
	Overall	104	104	-	26.9	21.9	35.6	53.80%	0.451	0.643	24.00%	0.171	0.338	3.80%	0.015	0.101
*HER2 + Early stage − 1st Line*																
OS from diagnosis	*PIK3CA* altered	40	19	0.896	163	101.9	NA	100.00%	1	1	75.80%	0.632	0.91	57.60%	0.425	0.78
	*PIK3CA* not altered	104	40		134.8	87.9	168.4	96.00%	0.923	0.999	78.50%	0.699	0.881	54.60%	0.432	0.689
	Overall	144	59	-	134.8	98.8	163.7	97.20%	0.944	0.999	77.70%	0.705	0.857	55.40%	0.46	0.668
OS from treatment	*PIK3CA* altered	40	NA	NA	NA	NA	NA	NA	NA	NA	NA	NA	NA	NA	NA	NA
	*PIK3CA* not altered	104	NA	NA	NA	NA	NA	NA	NA	NA	NA	NA	NA	NA	NA	NA
	Overall	144	NA	-	NA	NA	NA	NA	NA	NA	NA	NA	NA	NA	NA	NA
DRFS from diagnosis	*PIK3CA* altered	33	33	0.077	36.6	27.1	62.6	72.70%	0.59	0.896	33.30%	0.206	0.54	3.00%	0.004	0.209
	*PIK3CA* not altered	80	80		22.9	16.8	32.8	48.70%	0.389	0.61	18.70%	0.119	0.296	3.70%	0.012	0.114
	Overall	113	113	-	26.3	21.8	35.5	55.80%	0.473	0.657	23.00%	0.164	0.322	3.50%	0.014	0.093
DRFS from treatment	*PIK3CA* altered	31	31	0.16	35.4	27	61.7	71.00%	0.567	0.889	32.30%	0.194	0.537	3.20%	0.005	0.222
	*PIK3CA* not altered	71	71		22.4	19.3	33.5	46.50%	0.362	0.597	19.70%	0.123	0.315	4.20%	0.014	0.128
	Overall	102	102	-	26.9	22.3	35.4	53.90%	0.451	0.645	23.50%	0.166	0.334	3.90%	0.015	0.102
*HER2 + Early stage − 2nd line*																
OS from diagnosis	*PIK3CA* altered	40	20	0.867	152.9	101.9	NA	100.00%	1	1	76.40%	0.64	0.912	58.90%	0.44	0.787
	*PIK3CA* not altered	97	40		134.8	86.4	163.7	95.80%	0.918	0.999	77.50%	0.687	0.874	53.50%	0.421	0.679
	Overall	137	60	-	134.8	98.8	163	97.00%	0.942	0.999	77.20%	0.698	0.852	55.20%	0.458	0.665
OS from Treatment	*PIK3CA* altered	40	NA	NA	NA	NA	NA	NA	NA	NA	NA	NA	NA	NA	NA	NA
	*PIK3CA* not altered	97	NA	NA	NA	NA	NA	NA	NA	NA	NA	NA	NA	NA	NA	NA
	Overall	137	NA	-	NA	NA	NA	NA	NA	NA	NA	NA	NA	NA	NA	NA
DRFS from diagnosis	*PIK3CA* altered	33	33	0.058	38.9	28.6	72.9	72.70%	0.59	0.896	36.40%	0.232	0.571	3.00%	0.004	0.209
	*PIK3CA* not altered	78	78		24	16.8	33.3	50.00%	0.4	0.624	19.20%	0.122	0.303	3.80%	0.013	0.117
	Overall	111	111	-	28	23.4	36.6	56.80%	0.482	0.668	24.30%	0.175	0.338	3.60%	0.014	0.094
DRFS from treatment	*PIK3CA* altered	31	31	0.133	38.5	27.7	70.5	71.00%	0.567	0.889	35.50%	0.221	0.57	3.20%	0.005	0.222
	*PIK3CA* not altered	69	69		22.7	19.3	33.7	47.80%	0.374	0.612	20.30%	0.127	0.324	4.30%	0.014	0.131
	Overall	100	100	-	28.4	22.4	38.5	55.00%	0.461	0.657	25.00%	0.178	0.351	4.00%	0.015	0.104
*HER2 + Stage IV*																
OS from diagnosis	*PIK3CA* altered	15	7	0.595	114.8	53.1	NA	85.70%	0.692	1	55.60%	0.343	0.899	41.70%	0.198	0.876
	*PIK3CA* not altered	47	15		117.3	81.3	NA	100.00%	1	1	87.20%	0.772	0.986	41.60%	0.227	0.764
	Overall	62	22	-	114.8	81.3	NA	96.60%	0.922	1	78.60%	0.679	0.91	42.10%	0.261	0.677
OS from treatment	*PIK3CA* altered	15	7	0.369	112.1	51.1	NA	85.70%	0.692	1	55.60%	0.343	0.899	41.70%	0.198	0.876
	*PIK3CA* not altered	46	14		120.3	80.8	NA	95.30%	0.893	1	89.30%	0.796	1	52.20%	0.338	0.805
	Overall	61	21	-	112.1	80.8	NA	93.00%	0.867	0.999	79.90%	0.693	0.922	48.30%	0.323	0.723
PFS from diagnosis	*PIK3CA* altered	15	15	0.229	7.1	1.6	32.2	20.00%	0.073	0.55	6.70%	0.01	0.443	0.00%	NA	NA
	*PIK3CA* not altered	47	42		15	7.2	23.2	31.90%	0.21	0.485	12.00%	0.049	0.292	0.00%	NA	NA
	Overall	62	57	-	11.6	7.1	18.5	29.00%	0.197	0.428	10.30%	0.045	0.235	0.00%	NA	NA
PFS from treatment	*PIK3CA* altered	15	7	0.904	28.7	6.4	NA	50.60%	0.265	0.967	25.30%	0.055	1	0.00%	NA	NA
	*PIK3CA* not altered	44	20		14.2	7.9	NA	34.20%	0.189	0.62	19.00%	0.066	0.548	19.00%	0.066	0.548
	Overall	59	27	-	14.2	6.9	55.6	36.80%	0.225	0.602	20.70%	0.088	0.484	0.00%	NA	NA
*HER2 + Stage IV − 1st line*																
OS from diagnosis	*PIK3CA* altered	14	7	0.214	114.8	37.6	NA	84.60%	0.671	1	51.90%	0.303	0.891	34.60%	0.132	0.909
	*PIK3CA* not altered	44	13		120.9	81.3	NA	100.00%	1	1	88.80%	0.788	1	50.60%	0.32	0.799
	Overall	58	20	-	114.8	81.3	NA	96.40%	0.916	1	78.90%	0.678	0.917	44.30%	0.276	0.71
OS from treatment	*PIK3CA* altered	14	NA	NA	NA	NA	NA	NA	NA	NA	NA	NA	NA	NA	NA	NA
	*PIK3CA* not altered	44	NA	NA	NA	NA	NA	NA	NA	NA	NA	NA	NA	NA	NA	NA
	Overall	58	NA	-	NA	NA	NA	NA	NA	NA	NA	NA	NA	NA	NA	NA
PFS from diagnosis	*PIK3CA* altered	14	14	***0.042***	5.5	1.6	32.2	14.30%	0.04	0.515	0.00%	NA	NA	0.00%	NA	NA
	*PIK3CA* not altered	44	39		14.9	7.2	22	29.50%	0.187	0.466	12.80%	0.053	0.311	0.00%	NA	NA
	Overall	58	53	-	8.9	7.1	18.2	25.90%	0.167	0.4	8.90%	0.034	0.232	0.00%	NA	NA
PFS from treatment	*PIK3CA* altered	14	NA	NA	NA	NA	NA	NA	NA	NA	NA	NA	NA	NA	NA	NA
	*PIK3CA* not altered	44	NA	NA	NA	NA	NA	NA	NA	NA	NA	NA	NA	NA	NA	NA
	Overall	58	NA	-	NA	NA	NA	NA	NA	NA	NA	NA	NA	NA	NA	NA
*HER2 + Stage IV − 2nd line*																
OS from diagnosis	*PIK3CA* altered	13	7	0.254	114.8	37.6	NA	84.60%	0.671	1	51.90%	0.303	0.891	34.60%	0.132	0.909
	*PIK3CA* not altered	38	13		120.9	81.3	NA	100.00%	1	1	87.80%	0.772	1	50.00%	0.316	0.792
	Overall	51	20	-	114.8	81.3	NA	96.00%	0.908	1	77.60%	0.661	0.911	43.50%	0.271	0.701
OS from treatment	*PIK3CA* altered	13	NA	NA	NA	NA	NA	NA	NA	NA	NA	NA	NA	NA	NA	NA
	*PIK3CA* not altered	38	NA	NA	NA	NA	NA	NA	NA	NA	NA	NA	NA	NA	NA	NA
	Overall	51	NA	-	NA	NA	NA	NA	NA	NA	NA	NA	NA	NA	NA	NA
PFS from diagnosis	*PIK3CA* altered	13	13	0.147	3.9	1	NA	15.40%	0.043	0.55	0.00%	NA	NA	0.00%	NA	NA
	*PIK3CA* not altered	38	36		12	3	22	26.30%	0.155	0.448	7.90%	0.023	0.269	0.00%	NA	NA
	Overall	51	49	-	7.4	3.2	16.3	23.50%	0.143	0.386	5.50%	0.015	0.196	0.00%	NA	NA
PFS from treatment	*PIK3CA* altered	13	NA	NA	NA	NA	NA	NA	NA	NA	NA	NA	NA	NA	NA	NA
	*PIK3CA* not altered	38	NA	NA	NA	NA	NA	NA	NA	NA	NA	NA	NA	NA	NA	NA
	Overall	51	NA	-	NA	NA	NA	NA	NA	NA	NA	NA	NA	NA	NA	NA

*p*-values calculated using the log-rank test comparing *PIK3CA* Altered vs. Not Altered groups. Survival probabilities estimated using Kaplan-Meier method.

The bold *p*-values indicate statistical significance (*p* < 0.05)

**Table 3:** Univariable and multivariable Cox Proportional Hazard Models for Overall Survival after diagnosis for HER2 + patients

Characteristic		Univariable			Multivariable		
	*N*	HR	95% CI	*p*-value	aHR	95% CI	*p*-value
Age at diagnosis	212	1	1.01, 1.08	**0.015**	1.02	0.99, 1.06	0.2
Race	212	–	–	–	–	–	–
White	–	—	—	–	—	—	–
Black	–	1.4	0.68, 2.75	0.4	1.22	0.59, 2.54	0.6
Asian	–	0.7	0.21, 2.15	0.5	0.84	0.23, 3.01	0.8
Other/Unknown	–	0.7	0.26, 1.99	0.5	0.46	0.16, 1.34	0.2
Ethnicity	212	–	–	–	–	–	–
Non-Hispanic		–	–				
Hispanic	–	0.7	0.18, 2.98	0.7	–	–	–
Unknown	–	0.7	0.10, 5.18	0.7	–	–	–
Tumor Laterality	212	–	–	–	–	–	–
Left		–	–		–	–	
Other/Unknown	–	1.4	0.79, 2.49	0.3	1.64	0.87, 3.08	0.12
Right	–	0.8	0.47, 1.25	0.3	0.77	0.46, 1.29	0.3
Histology	212	—	—	—	—	—	—
Invasive ductal carcinoma	—	—	—	—	—	—	—
Invasive lobular carcinoma	—	2.5	0.77, 7.84	0.13	2.09	0.63, 6.88	0.2
Other histology	—	4.5	1.10, 18.7	**0.036**	4.29	0.89, 20.7	0.069
ER Status	212	—	—	—	—	—	—
Positive	—	—	—				
Negative	—	2.2	1.44, 3.49	**< 0.001**			
Borderline							
Unknown/Not tested							
PR Status	212						
Positive		—	—		—	—	
Negative		2.2	1.45, 3.46	**< 0.001**	1.7	0.69, 4.19	0.2
Unknown/Not tested		0	0.00, Inf	> 0.9	0	0.00, Inf	> 0.9
Stage at diagnosis	212						
Early stage (I-III)		—	—				
Stage IV		1.2	0.72, 1.93	0.5			
Unknown/Other stage							
Breast cancer subtype	212						
HER2+, HR-		—	—		—	—	
HER2+, HR+		0.4	0.26, 0.64	**< 0.001**	1.43	0.50, 4.08	0.5
Received chemotherapy	212						
Yes		—	—				
No		2.2	0.55, 9.23	0.3			
Received hormone therapy	212						
Yes		—	—		—	—	
No		2.8	1.76, 4.46	**< 0.001**	3.23	1.55, 6.71	**0.002**
Received immunotherapy	212						
Yes		—	—		—	—	
No		0.4	0.23, 0.76	**0.004**	0.55	0.29, 1.03	0.062
Received HER2 therapy	212						
Yes		—	—		—	—	
No		0.6	0.27, 1.13	0.1	0.31	0.12, 0.76	**0.011**
Received CDK4/6 inhibitors	212						
Yes		—	—				
No		1.3	0.77, 2.32	0.3			
Received PI3K inhibitors	212						
Yes		—	—				
No		1.3	0.67, 2.41	0.5			
Received other therapy	212						
Yes		—	—				
No		2.3	0.73, 7.40	0.2			
TP53 alteration	212						
Altered		—	—				
Not altered		0.7	0.41, 1.03	0.068			
*PIK3CA* alteration	212	1.1	0.68, 1.66	0.8			
ERBB2 alteration	212						
Altered		—	—		—	—	
Not altered		1.3	0.80, 1.99	0.3	2.21	1.25, 3.91	**0.006**
p53 pathway	212						
Altered		—	—				
Not altered		0.7	0.44, 1.20	0.2			
PI3K pathway	212						
Altered		—	—				
Not altered		1.3	0.83, 1.98	0.3			
Cell cycle pathway	212						
Altered		—	—				
Not altered		0.9	0.55, 1.39	0.6			

CI, Confidence interval; HR, Hazard ratio; aHR, adjusted Hazard Ratio

The bold *p*-values indicate statistical significance (*p* < 0.05)

The median OS from treatment (Table 2) was 125.1 months (95% CI, 98.3–155.3) for the overall cohort: 131.2 months (95% CI, 98.3–160.6) for early-stage patients, and 112.1 months (95% CI, 80.8–NA) for those with stage IV disease. Univariable COX PH analysis showed that *PIK3CA*m was not associated with OS (HR = 1.11, 95% CI 0.70–1.75; Table 4). LASSO did not select *PIK3CA*m for the set of covariates in multivariable COX PH analysis (Table 4). Similar results were found in subgroup analyses by stage and by ER-status (Supplemental Tables 11–14).

**Table 4:** Univariable and multivariable Cox Proportional Hazard Models for Overall Survival after treatment for HER2 + patients

Characteristic		Univariable			Multivariable		
	*N*	HR	95% CI	*p*-value	aHR	95% CI	*p*-value
Age at Diagnosis	209	1.04	1.00, 1.07	**0.026**	1.02	0.99, 1.06	0.2
Race	209						
White		—	—				
Black		1.4	0.68, 2.77	0.4			
Asian		0.7	0.22, 2.30	0.6			
Other/Unknown		0.9	0.31, 2.35	0.8			
Ethnicity	209						
Non-Hispanic		—	—				
Hispanic		0.8	0.19, 3.16	0.7			
Unknown		1.1	0.16, 8.23	> 0.9			
Tumor Laterality	209						
Left		—	—		—	—	
Other/Unknown		1.1	0.58, 2.03	0.8	1.24	0.63, 2.43	0.5
Right		0.8	0.46, 1.23	0.3	0.75	0.45, 1.24	0.3
Histology	209						
Invasive ductal carcinoma		—	—		—	—	
Invasive lobular carcinoma		2.5	0.79, 8.08	0.12	1.97	0.59, 6.51	0.3
Other histology		4.8	1.17, 19.9	**0.03**	5.29	1.12, 25.0	**0.035**
ER Status	209						
Positive		—	—				
Negative		1.9	1.20, 2.91	**0.006**			
Borderline							
Unknown/Not tested							
PR Status	209						
Positive		—	—		—	—	
Negative		2	1.32, 3.15	**0.001**	1.07	0.59, 1.95	0.8
Unknown/Not tested		0	0.00, Inf	> 0.9	0	0.00, Inf	> 0.9
Stage at Diagnosis	209						
Early stage (I-III)		—	—				
Stage IV		1	0.60, 1.67	> 0.9			
Unknown/Other stage							
Breast cancer subtype	209						
HER2+, HR-		—	—				
HER2+, HR+		0.5	0.30, 0.74	**0.001**			
Received chemotherapy	209						
Yes		—	—				
No		0	0.00, Inf	> 0.9			
Received hormone therapy	209						
Yes		—	—		—	—	
No		2.8	1.73, 4.50	**< 0.001**	2.55	1.33, 4.86	**0.005**
Received immunotherapy	209						
Yes		—	—		—	—	
No		0.4	0.24, 0.79	**0.006**	0.48	0.25, 0.91	**0.025**
Received HER2 therapy	209						
Yes		—	—		—	—	
No		0.5	0.21, 1.05	0.064	0.4	0.16, 0.99	**0.048**
Received CDK4/6 inhibitors	209						
Yes		—	—		—	—	
No		1.5	0.84, 2.62	0.2	1.73	0.85, 3.53	0.13
Received PI3K inhibitors	209						
Yes		—	—				
No		1.5	0.74, 2.83	0.3			
Received other therapy	209						
Yes		—	—				
No		2.2	0.68, 6.88	0.2			
TP53 alteration	209						
Altered		—	—				
Not altered		0.6	0.40, 1.01	0.056			
*PIK3CA* alteration	209	1.1	0.70, 1.75	0.7			
ERBB2 alteration	209						
Altered		—	—		—	—	
Not altered		1.2	0.75, 1.92	0.4	2.27	1.23, 4.16	**0.008**
p53 pathway	209						
Altered		—	—				
Not altered		0.8	0.47, 1.30	0.3			
PI3K pathway	209						
Altered		—	—				
Not altered		1.2	0.77, 1.86	0.4			
Cell cycle pathway	209						
Altered		—	—				
Not altered		0.9	0.58, 1.50	0.8			

CI, Confidence interval; HR = Hazard ratio; aHR = adjusted hazard ratio

The bold *p*-values indicate statistical significance (*p* < 0.05)

The median DRFS and PFS from initial BC diagnosis (Table 2) were 26.3 months (95% CI, 21.8–35.5) and 11.6 months (95% CI, 7.1–18.5), respectively. Univariable COX PH analysis showed that *PIK3CA*m was not associated with a higher risk of distant recurrence (HR = 0.67, 95% CI 0.44–1.01) or progression (HR = 1.44, 95% CI 0.79–2.63); Supplemental Tables 15 and 16). *PIK3CA*m was not selected by LASSO for the multivariable analysis.

The median DRFS and PFS from the time of first treatment (Table 2) were 26.9 months (95% CI 21.9–35.6) and 14.2 months (95% CI 6.9–55.6), respectively. Univariable COX PH analysis showed that *PIK3CA*m was not associated with distant recurrence (HR = 0.7, 95% CI 0.44–1.01; Table 5) or progression (HR = 0.95, 95% CI 0.38–2.36; Table 6). LASSO did not select *PIK3CA*m for multivariable analyses (Tables 5 and 6).

**Table 5:** Univariable and multivariable Cox Proportional Hazard Models for Distant Recurrence-Free Survival after treatment for Early Stage HER2 + patients

Characteristic		Univariable			Multivariable		
	*N*	HR	95% CI	*p*-value	aHR	95% CI	*p*-value
Age at Diagnosis	115	1.1	1.02, 1.08	**0.002**	1.03	1.00, 1.06	0.053
Race	115						
White		—	—				
Black		1.2	0.65, 2.31	0.5			
Asian		0.5	0.21, 1.32	0.2			
Other/Unknown		1.6	0.91, 2.89	0.1			
Ethnicity	115						
Non-Hispanic		—	—				
Hispanic		0.9	0.29, 2.93	0.9			
Unknown		1.3	0.42, 4.17	0.6			
Tumor Laterality	115						
Left		—	—				
Other/Unknown		1.4	0.81, 2.28	0.3			
Right		1	0.63, 1.43	0.8			
Histology	115						
Invasive ductal carcinoma		—	—				
Invasive lobular carcinoma		2.8	0.68, 11.6	0.2			
Other histology		1.4	0.34, 5.65	0.7			
ER Status	115						
Positive		—	—				
Negative		2.2	1.48, 3.34	**< 0.001**			
Borderline							
Unknown/Not Tested							
PR Status	115						
Positive		—	—				
Negative		1.9	1.28, 2.78	**0.001**			
Unknown/Not tested		1.6	0.22, 11.8	0.6			
Breast cancer subtype	115						
HER2+, HR-		—	—				
HER2+, HR+		0.5	0.30, 0.68	**< 0.001**			
Received hormone therapy	115						
Yes		—	—		—	—	
No		2.9	1.84, 4.43	**< 0.001**	2.1	1.31, 3.37	**0.002**
Received immunotherapy	115						
Yes		—	—				
No		0.6	0.38, 1.07	0.086			
Received HER2 therapy	115						
Yes		—	—		—	—	
No		0.4	0.18, 0.68	**0.002**	0.5	0.25, 1.03	0.059
Received CDK4/6 inhibitors	115						
Yes		—	—		—	—	
No		2.1	1.28, 3.28	**0.003**	1.43	0.85, 2.40	0.2
Received PI3K inhibitors	115						
Yes		—	—				
No		1.3	0.72, 2.29	0.4			
Received other therapy	115						
Yes		—	—				
No		1.4	0.86, 2.38	0.2			
TP53 alteration	115						
Altered		—	—				
Not altered		0.6	0.42, 0.92	**0.017**			
*PIK3CA* Alteration	115	0.7	0.44, 1.00	0.052			
ERBB2 Alteration	115						
Altered		—	—				
Not altered		0.7	0.46, 1.04	0.074			
p53 pathway	115						
Altered		—	—				
Not altered		0.6	0.36, 0.87	**0.01**			
PI3K pathway	115						
Altered		—	—				
Not altered		1.4	0.96, 2.02	0.079			
Cell cycle pathway	115						
Altered		—	—				
Not altered		1.1	0.76, 1.66	0.6			

CI, Confidence interval; HR, Hazard ratio; aHR, adjusted Hazard Ratio

The bold *p*-values indicate statistical significance (*p* < 0.05)

**Table 6:** Univariable and multivariable Cox Proportional Hazard Models for Progression Free Survival after treatment for Stage IV HER2 + patients

Characteristic		Univariable			Multivariable		
	*N*	HR	95% CI	*p*-value	aHR	95% CI	*p*-value
Age at Diagnosis	59	1	0.97, 1.09	0.3			
Race	59						
White		—	—				
Black		0.5	0.07, 3.97	0.5			
Asian		2.9	0.66, 13.1	0.2			
Other/Unknown		2.1	0.26, 16.3	0.5			
Ethnicity	59						
Non-Hispanic		—	—		—	—	
Hispanic		1.1	0.14, 8.10	> 0.9	1.13	0.15, 8.54	> 0.9
Unknown		56	3.47, 890	**0.005**	71.6	4.46, 1,150	**0.003**
Tumor Laterality	59						
Left		—	—				
Other/Unknown		0.7	0.23, 2.21	0.6			
Right		0.8	0.35, 1.93	0.6			
Histology	59						
Invasive ductal carcinoma		—	—				
Invasive lobular carcinoma		1.3	0.30, 5.49	0.7			
Other histology							
ER Status	59						
Positive		—	—				
Negative		1	0.45, 2.20	> 0.9			
Borderline							
Unknown/Not tested							
PR Status	59						
Positive		—	—				
Negative		1.3	0.59, 2.76	0.5			
Unknown/Not tested							
Breast cancer subtype	59						
HER2+, HR-		—	—				
HER2+, HR+		0.9	0.40, 1.96	0.8			
Received chemotherapy	59						
Yes		—	—				
No		0	0.00, Inf	> 0.9			
Received hormone therapy	59						
Yes		—	—				
No		1	0.46, 2.19	> 0.9			
Received immunotherapy	59						
Yes		—	—		—	—	
No		0.1	0.02, 0.43	**0.002**	0.08	0.02, 0.38	**0.001**
Received HER2 therapy	59						
Yes		—	—		—	—	
No		0	0.00, Inf	> 0.9	0	0.00, Inf	> 0.9
Received CDK4/6 inhibitors	59						
Yes		—	—				
No		1	0.22, 4.15	> 0.9			
Received PI3K inhibitors	59						
Yes		—	—				
No		2.6	0.34, 19.1	0.4			
Received other therapy	59						
Yes		—	—				
No		0.5	0.13, 1.64	0.2			
TP53 alteration	59						
Altered		—	—				
Not altered		0.8	0.33, 1.96	0.6			
*PIK3CA* Alteration	59	1	0.38, 2.36	> 0.9			
ERBB2 Alteration	59						
Altered		—	—				
Not altered		1.4	0.47, 4.09	0.5			
p53 pathway	59						
Altered		—	—				
Not altered		1	0.38, 2.39	> 0.9			
PI3K pathway	59						
Altered		—	—				
Not altered		1.7	0.70, 3.97	0.3			
Cell cycle pathway	59						
Altered		—	—				
Not altered		0.9	0.32, 2.30	0.8			

CI, Confidence interval; HR, Hazard ratio; aHR, adjusted Hazard Ratio

The bold *p*-values indicate statistical significance (*p* < 0.05)

### *Survival outcomes on patients with PIK3CA*m

The median OS from initial diagnosis was 126 months (95% CI 98.8-NA; Table 2) for those with *PIK3CA*m. For these patients, none of the covariates selected for the multivariable model were associated with worse OS (Supplemental Table 17). On subgroup analysis by 1st or 2nd line of therapy (Supplemental Table 17), not having received hormone therapy was associated with worse survival (aHR = 4.65, 95% CI 1.78–12.1 for 1st line and aHR = 4.97, 95% CI 1.91–12.9 for 2nd line). There were not enough patients to stratify analysis by stage and ER-status.

The median OS from treatment was 125.1 months (95% CI 98.3-NA; Table 2) for those with a *PIK3CA*m. For these patients, as expected, not having received HER2-therapy was associated with worse survival (aHR = 4.51, 95% CI 1.77–11.5; Supplemental Table 18). On subgroup analysis by 1st or 2nd line of therapy (Supplemental Table 18), not having received hormone therapy was again associated with worse survival (aHR = 4.67, 95% CI 1.79–12.2 for 1st line and aHR = 5.00, 95% CI 1.89–13.3 for 2nd line). Patient numbers were insufficient for stratification by stage and ER-status.

Among patients with early-stage disease, *PIK3CAm* was associated with longer DRFS from diagnosis (37.7 months [95% CI 28.6–62.6] for *PIK3CAm* vs. 22.0 months [95% CI 16.8–32.8] for *non-PIK3CAm*; *p* = 0.04). In contrast, among patients with Stage IV disease receiving first-line therapy, *PIK3CAm* was associated with shorter median PFS (5.5 months [95% CI 1.6–32.2] for PIK3CAm vs. 14.9 months [95% CI 7.2–22.0] for *non-PIK3CAm*; *p* = 0.04). The median DRFS from treatment for those with *PIK3CA*m (Table 2) was 36.9 months (95% CI 27.7–61.7). There were not enough patients and/or follow-up to obtain PFS from treatment on patients with *PIK3CA*m.

## Discussion

In this retrospective cohort study that utilized real-world data from the GENIE BPC, we evaluated the clinical impact of *PIK3CA*m in 212 HER2 + BC patients receiving first- and second-line therapies. Our primary findings revealed that *PIK3CA*m was not significantly associated with worse OS, PFS, or distant-recurrence risk, according to COX PH regressions. However, Stage IV HER2 + patients with *PIK3Ca*m receiving first-line therapy had a statistically lower PFS with first line therapy compared to wild-type *PIK3CA* (median PFS from diagnosis: 5.5 months for *PIK3CA*m vs. 14.9 months for wild-type, *p* = 0.04). In addition to the main findings, patients with *PIK3CA*m in this cohort had higher rates of ER-positive disease (78% vs. 56%, *p* = 0.007) and lower rates of receiving HER2-targeted therapies (84% vs. 94%, *p* = 0.04) compared to those with wild-type *PIK3CA*. Interestingly, early-stage patients with *PIK3CA*m also showed a longer median time from diagnosis to distant metastasis (38 months vs. 22 months, *p* = 0.01) compared to those without.

Prior studies have reported associations between *PIK3CAm* and worse outcomes in HER2 + BC [15, 23]. In early-stage HER2 + BC, the NeoALTTO, GeparSixto, and NeoSphere trials reported up to a 20% decrease in pCR rates in *PIK3CAm* versus wild-type patients after neoadjuvant therapy with HER2-targeted agents, while the APHINITY trial showed poorer clinical outcomes after adjuvant trastuzumab and pertuzumab in patients with PIK3 alterations [24–27]. A meta-analysis reported that *PIK3CAm* was associated with lower probability of pCR (OR 0.23, 95% CI 0.19–0.27) in HER2 + BC treated with anti-HER2 therapy in the neoadjuvant setting, independent of single- or dual-agent anti-HER2 therapy [23]. The same meta-analysis found no impact of *PIK3CAm* on disease free survival in the neoadjuvant (HR 1.19, 95% CI 0.85–1.63) or adjuvant (HR 0.84, 95% CI 0.66–1.08) settings, or on invasive disease-free survival (HR 1.24, 95% CI 0.93–1.65), although only a few studies contributed to these outcomes [23]. In HER2 + mBC patients, *PIK3CAm* was also not associated with worse OS (HR 1.29, 95% CI 0.79–2.11), consistent with our findings (23). Regarding PFS, *PIK3CAm* was associated with a shorter PFS among patients treated with docetaxel, trastuzumab, and pertuzumab or capecitabine plus lapatinib in CLEOPATRA and EMILIA, respectively [28–30]. Conversely, ExteNET and KATHERINE showed no impact of *PIK3CAm* in early-stage HER2 + BC receiving adjuvant neratinib or T-DM1, and EMILIA and TH3RESA reported no impact of *PIK3CAm* on PFS in HER2 + mBC treated with T-DM1 [31–33].

The observed discordance between significantly shorter PFS among patients receiving first-line therapy and the lack of OS differences in Stage IV PIK3CAm in our study may reflect the fact that patients who progress on one therapy can subsequently benefit from other effective therapies, thereby extending their overall lifespan [34]. The continuously evolving therapeutic landscape for HER2 + BC, including advanced antibody-drug conjugates and tyrosine kinase inhibitors, may mitigate the early negative impact of *PIK3CA* mutations on long-term survival in this patient population, allowing for effective disease management post-progression [5, 35]. In addition, despite *PIK3CA* being a known oncogenic driver, its limited impact on OS in our cohort, especially when stratified by TNM stage, underscores the dominant prognostic role of TNM staging, which can supersede the influence of individual molecular alterations like *PIK3CA*m, particularly in a context of effective sequential treatment options [36]. While *PIK3CA*m may signal a more aggressive tumor biology that may lead to earlier progression, the ability to manage the disease through subsequent lines of therapy may attenuate a potential negative effect on OS in HER2 + BC.

Within the *PIK3CA*m cohort analyzed, the association of not having received hormone therapy with worse OS highlights the critical role of hormone receptor signaling in influencing long-term survival, even in HER2 + disease, and underscores the importance of endocrine therapy where applicable [37–39]. In contrast, our finding that other concurrent mutations were not associated with worse survival in patients with *PIK3CA*m contrasts with results from a large analysis of over 5,600 mBC patients, in which 42% of those with ERBB2 mutations also had a concurrent *PIK3CA*m, and the presence of an ERBB2 mutation in this population was associated with a better clinical response to anti-HER2 targeted therapies [40]. Additionally, the finding that patients with *PIK3CA*m received lower rates of HER2-targeted therapy aligns with evidence suggesting that *PIK3CA*m may confer resistance to agents such as trastuzumab and lapatinib [15, 23, 41, 42]. However, the biological mechanisms underlying increased resistance to HER2 therapy in the presence of *PIK3CAm* remain poorly understood, and current evidence on this relationship is inconclusive [15, 23, 42]. Conversely, our observation that *PIK3CA*m patients exhibited higher rates of ER-positive disease is consistent with prior literature, which consistently reports *PIK3CA* mutations as more prevalent in ER-positive tumors [8, 11, 43, 44].

One interesting finding was the lower rates of Hippo pathway alterations in patients with *PIK3CA*m. The Hippo pathway, one of the most frequently dysregulated signaling pathways in human cancer, is a kinase cascade that regulates cell proliferation, differentiation, and survival [45]. In cancer, it plays a critical role in controlling contact-dependent cell growth and proliferation [45–48]. YAP and TAZ, key downstream effectors of the Hippo pathway, are activated following damage to various organs, playing roles in tissue regeneration and wound healing [45]. The Hippo pathway has also been implicated in metastasis, with evidence showing that components such as LAT1/2, MST1/2, YAP, and TAZ influence tumor spread [45, 49]. In most contexts, Hippo signaling acts as an inhibitor of metastasis [5]. Another important role of this pathway is in treatment resistance, with studies suggesting that resistance to systemic therapies used in BC can be reversed by targeting Hippo pathway elements [45, 47]. The relationship between Hippo signaling and *PIK3CA*m may lie in the fact that YAP and TAZ drive epithelial cell proliferation in response to both mechanical stimuli and growth factor signaling through the PI3K-PDK1-Akt pathway [50]. Our observation of longer time to distant metastasis in patients with *PIK3CA*m may be explained by the lower frequency of Hippo pathway alterations in this group, given the pathway’s role in metastasis. A possible hypothesis for these lower rates of Hippo alteration is mutual exclusivity, the principle that alterations affecting the same biological pathway tend not to co-occur within the same patient [51]. Finally, these lower rates of Hippo alterations may also relate to resistance to HER2-targeted therapies in *PIK3CA*m, as inhibition of Hippo signaling has been shown to mitigate treatment resistance.

From a clinical practice perspective, despite the evidence linking *PIK3CA*m with poor outcomes in HER2 + BC patients, and despite clear evidence that *PIK3CA*m drives resistance to standard HER2-targeted therapies by maintaining PI3K pathway activation, *PIK3CA* mutation testing isn’t currently routinely integrated into clinical decision-making for HER2 + patients, and no PI3K pathway inhibitors are currently FDA-approved specifically for HER2 + BC [15, 30, 41]. Given that *PIK3CA*m Stage IV HER2 + patients receiving first-line therapy had a lower PFS, our study reinforces that routine *PIK3CA* mutation testing at diagnosis for HER2 + patients could be explored as a potential tool to risk-stratify patients and guide future personalized treatment strategies. While currently approved PI3K inhibitors are primarily for HR+/HER2- BC, our real-world findings provide further rationale for evaluating such agents in clinical trials for HER2 + *PIK3CAm* patients who exhibit resistance or rapid progression on standard HER2-directed therapies. There are several ongoing clinical trials currently addressing this question (Supplemental Table 19).

Our study has several limitations. The use of retrospective data can introduce issues of data quality, completeness, and confounding [52]. The GENIE BPC dataset has documented differences compared to other population-based registries [53, 54]. Studies have shown that patients in GENIE BPC colorectal cancer cohort tend to be younger, present with more advanced disease, have a greater prevalence of aggressive subtypes like triple-negative BC, and receive chemotherapy more frequently, often resulting in lower overall survival rates compared to SEER populations [54]. These inherent characteristics of the GENIE BPC cohort may limit the generalizability of our findings to the broader HER2 + BC population and necessitate careful interpretation when extrapolating results. The observation that some patients with *PIK3CAm* HER2 + BC did not receive standard anti-HER2 therapy likely reflects inter-institutional variability and provider-level differences, indicating that the data may contain heterogeneity and real-world nuances due to the multi-institutional nature of the dataset. Furthermore, the small number of patients in the *PIK3CA*m subgroup (*n* = 58) and subsequent subgroup analyses can reduce statistical power and lead to wide confidence intervals for certain estimates, potentially impacting the robustness of some conclusions. This may also explain the discrepancy in p-values observed between the log-rank and Cox regression analyses for DRFS. The limited sample sizes in several subgroup analyses (e.g., by line of therapy, stage, and ER status within the *PIK3CA*m group) resulted in insufficient patient numbers for robust multivariable modeling, precluding us from fully elucidating independent predictors in these specific contexts. Some patients began therapy prior to final diagnostic confirmation, which caused the lower median OS observed from diagnosis compared with OS from treatment initiation. On the other side, the use of a large, multi-institutional, real-world database like GENIE BPC provides valuable insights into clinical practice and complements findings from highly controlled clinical trials by reflecting actual patient management and outcomes in diverse clinical settings. The integration of comprehensive clinical and genomic data allows for a holistic evaluation of disease characteristics and treatment outcomes. The rigorous quality control and assurance protocols employed by GENIE BPC enhance the reliability of the data, and our use of advanced statistical methods like LASSO regression helped to manage collinearity and select key variables in a data-driven manner where sufficient power existed.

In conclusion, our findings underscore the impact of *PIK3CA*m in HER2 + BC, especially in the metastatic setting. It could be explored as an important biomarker for identifying patients at a higher risk of disease progression and those who may be more resistant to standard HER2-targeted therapies. This necessitates the consideration of personalized treatment strategies, potentially including PI3K pathway inhibitors or alternative regimens, and emphasizes the need for enhanced monitoring for disease progression in this patient subgroup. Future prospective studies with larger, multicentric, more diverse cohorts are warranted to validate and expand these findings. Additionally, deeper bioinformatic mining of genomic data from Whole Exome/Genome Sequencing, starting with the raw Variant Call Format files, could help elucidate the underlying pathway-level mechanisms driving these clinical observations.

## Supplementary Information

Supplementary Material 1

## Data Availability

Data from AACR Project GENIE are available upon request. Access can be obtained through the following platforms: (1) Synapse Platform (SageBionetworks): https://synapse.org/genie; (2) cBioPortal for Cancer Genomics (MSK): https://www.cbioportal.org/genie/.
