# Supplementary material for: Impact of somatic PIK3CA mutations on clinical outcomes in HER2-positive breast cancer
Source: Breast Cancer Res. 2026 Jun 20;28:162. doi: 10.1186/s13058-026-02324-6 (PMC13617687; doi:10.1186/s13058-026-02324-6)
Supplement: Supplementary file 1 [file 13058_2026_2324_MOESM1_ESM.docx]

**Supplemental Material**

**Supplemental Table 1.** Cross-reference of datasets and covariates from GENIE BPC used to construct the final dataset for this study, including original categories and any re-categorizations applied.

| **Variable Original Dataset** | **Variable Original Name** | **Variable Description** | **Variable Original Nature** | **Variable Original Categories** | **Recategorized (New Categories)** |
| --- | --- | --- | --- | --- | --- |
| **Patient Characteristics & Demographics** | | | | | |
| Patient-level dataset | naaccr_ethnicity_code | Patient's ethnicity based on NAACCR coding. | Categorical | Non-Spanish; non-Hispanic, Mexican (includes Chicano), Puerto Rican, Cuban, South or Central American (except Brazil), Other specified Spanish/Hispanic origin (includes European; excludes Dominican Republic), Spanish NOS or Hispanic NOS or Latino NOS, Spanish surname only, Dominican Republic, Unknown whether Spanish or not | Non-Hispanic, Hispanic, Unknown |
| Patient-level dataset | naaccr_race_code_primary | Patient's primary race based on NAACCR coding. | Categorical | White, Black, American Indian, Aleutian, or Eskimo, Chinese, Japanese, Filipino, Hawaiian, Korean, Vietnamese, Laotian, Hmong, Kampuchean (Cambodian), Thai, Asian Indian or Pakistani NOS, Asian Indian, Pakistani, Micronesian NOS, Chamorro/Chamoru, Guamanian NOS, Polynesian NOS, Tahitian, Samoan, Tongan, Melanesian NOS, Fiji Islander, New Guinean, Other Asian, Pacific Islander NOS, Other, Unknown | White, Black, Asian (combining all Asian categories), Other/Unknown (combining remaining unspecified categories) |
| Patient-level dataset | naaccr_sex_code | Patient's sex based on NAACCR coding. | Categorical | Male, Female, Other intersex, disorders of sexual development/DSD, Transsexual NOS, Transsexual natal male, Transsexual natal female | Male, Female, Other/Unknown |
| Cancer diagnosis dataset | age_dx | Patient's age in years at the time of cancer diagnosis. | Numeric | Continuous (Years) | No recategorization |
| **Tumor Characteristics** | | | | | |
| Cancer diagnosis dataset | ca_seq | Sequence identifier for the patient's cancer diagnosis (0 = first and only, 1 = first of 2+, etc.). | Numeric | 0, 1, 2, ...10 | First and Only Primary (0), First of 2+ Primaries (1), Second or Subsequent Primary (>=2), Unknown |
| Cancer diagnosis dataset | ca_hist_brca | Histology of the breast cancer. | Categorical | Invasive lobular carcinoma, Invasive ductal carcinoma, Other histology | No recategorization |
| Cancer diagnosis dataset | naaccr_laterality_cd | Tumor laterality code (e.g., right, left, bilateral). | Numeric | 0, 1, 2, 3, 4, 5, 9 | Right (1), Left (2), Bilateral (4), Other/Unknown (all other codes) |
| Cancer diagnosis dataset | stage_dx | Derived stage of cancer at diagnosis (AJCC equivalent). | Character | Stage 0, Stage I, Stage II, Stage III, Stage I-III NOS, Stage IV | No recategorization |
| Cancer diagnosis dataset | ca_bca_er | Estrogen Receptor (ER) summary status for breast cancer. | Character | Positive/elevated, Negative/normal, Borderline, Not applicable, Test ordered/results not interpretable, Test ordered/results not in chart, Test not done, Unknown or no information | Positive, Negative, Borderline, Unknown/Not Tested |
| Cancer diagnosis dataset | ca_bca_pr | Progesterone Receptor (PR) summary status for breast cancer. | Character | Positive/elevated, Negative/normal, Borderline, Not applicable, Test ordered/results not interpretable, Test ordered/results not in chart, Test not done, Unknown or no information | Positive, Negative, Unknown/Not Tested |
| Cancer diagnosis dataset | ca_bca_her_summ | HER2 summary result for breast cancer. | Character | Positive/elevated/amplified, Negative/normal within normal limits, Borderline/equivocal/indeterminant, Not applicable, Test ordered results not in chart, Tests not done, Unknown or no information | Positive, Negative, Borderline, Unknown/Not Tested |
| Cancer diagnosis dataset | bca_subtype | Defined breast cancer molecular subtype (e.g., Triple Negative, HER2+). | Character | Triple Negative, HER2+, HR+, HER2+, HR-, HER2-, HR+ | Treated as factor. Missing values mapped to Unknown |
| **Treatment & Clinical History** | | | | | |
| Cancer diagnosis dataset | ca_tx_pre_path_stage | Indicates if neoadjuvant therapy (chemo/radiation) was given before pathologic staging. | Character | Yes, No, Not Applicable, Unknown | Yes, No, Unknown/Not Applicable (combining Not Applicable, Unknown, and empty strings) |
| Cancer diagnosis dataset | ca_n_regimens | Total number of cancer-directed regimens curated for the patient. | Numeric | Discrete (Number of regimens) | Treated as categorical |
| Cancer-directed regimen dataset | regimen_number | The sequence number of a cancer-directed regimen (e.g., 1st, 2nd). | Numeric | 1, 2, ...n | Treated as categorical in some contexts (e.g., for line of therapy), as numeric in others |
| Cancer-directed regimen dataset | drugs_num | Number of cancer-directed drugs in a specific regimen. | Numeric | 5-Jan | Treated as categorical |
| Cancer-directed regimen dataset | drugs_ct_yn | Indicates if the regimen was part of a clinical trial. | Character | Yes, No | Treated as factor |
| Cancer-directed regimen dataset | dx_reg_start_int_mos | Time in months from cancer diagnosis to the start of a regimen. | Numeric | Continuous (Months) | No recategorization |
| Cancer-directed regimen dataset | reg_start_end_all_int | Duration in days from the start to the end of all drugs in a regimen. | Numeric | Continuous (Days) | No recategorization |
| PRISSMM Imaging level dataset | dx_ref_scan_days | Time in days from first cancer diagnosis to a reference imaging date. | Numeric | Continuous (Days) | No recategorization |
| PRISSMM Imaging level dataset | image_scan_type | Type of imaging scan performed. | Character | CT, MRI, PET or PET-CT, Bone Scan, Other Nuclear Medicine Scan, Mammogram - Use for Breast Cancer only, Other CA-specific scan | Used to derive binary indicators: had_ct, had_mri, had_pet, had_bone_scan, had_mammogram (Yes/No) |
| PRISSMM Pathology level dataset | dx_path_proc_days | Time in days from first cancer diagnosis to a pathology procedure date. | Numeric | Continuous (Days) | No recategorization |
| PRISSMM Pathology level dataset | n_path_reports_pt | Total number of pathology reports for the patient. | Numeric | Discrete (Number of reports) | No recategorization |
| PRISSMM Pathology level dataset | pdl1_sum | Summary assessment of PD-L1 expression. | Character | High, Low, Positive, Negative, Indeterminate/Not stated | No recategorization, but only the latest value was used in some summaries |
| PRISSMM Medical Oncologist Assessment level dataset | dx_md_visit_days | Time in days from first cancer diagnosis to a medical oncologist visit. | Numeric | Continuous (Days) | No recategorization |
| PRISSMM Tumor Marker level dataset | tm_type | Type of tumor marker lab test performed. | Character | CA15-3, CA19-9, CA2729, CEA, PSA, Testosterone | Used to filter for specific tumor markers (CA15-3, CA2729), and then their median values (tm_num_result) were used |
| Cancer diagnosis dataset | ca_first_dmets1 | Primary site of distant metastasis at cancer diagnosis. | Character | ICD-O-3 topography code (various sites) | Used to derive specific distant metastasis sites at diagnosis (e.g., dmets_abdomen, dmets_bone, dmets_brain, dmets_liver, dmets_pelvis, dmets_thorax, dmets_trunk, dmets_extremity, dmets_head_neck, dmets_heme, dmets_breast), which are then treated as binary (Yes/No) |
| **Genomic & Sequencing Data** | | | | | |
| Cancer panel test level dataset | cpt_genie_sample_id | Unique GENIE Sample ID for the cancer panel test. | Character | GENIE Sample ID | Used to link to gene and pathway data; not directly used as a variable itself. |
| Cancer panel test level dataset | cpt_seq_date | Year of next generation sequencing. | Character | Year of sequencing (e.g., 2018, 2019) | Treated as categorical (Year of Sequencing) |
| Cancer panel test level dataset | cpt_report_post_death | Indicates if the NGS test report was returned on or after date of death. | Numeric | 1 (Yes), 0 (No) | Yes, No |
| Cancer panel test level dataset | dx_cpt_rep_days | Time in days from diagnosis to the sequencing report date. | Numeric | Continuous (Days) | No recategorization |
| Cancer panel test level dataset | dx_path_proc_cpt_days | Time in days from diagnosis to the pathology procedure corresponding to the NGS report. | Numeric | Continuous (Days) | No recategorization |
| Cancer panel test level dataset | path_proc_cpt_rep_days | Time in days from pathology procedure to the cancer panel test report date. | Numeric | Continuous (Days) | No recategorization |
| Cancer panel test level dataset | cpt_oncotree_code | OncoTree diagnosis code for the sequenced specimen. | Character | Various OncoTree codes (e.g., BA, BRCA, IDC) | Recoded to more descriptive terms (e.g., Breast Adenocarcinoma, Breast Invasive Carcinoma, Invasive Ductal Carcinoma, etc.) |
| Gene Alteration Data (Derived) | TP53, *PIK3CA*, ERBB2 | Alteration status for specific genes (e.g., TP53, *PIK3CA*, ERBB2). | Binary | 1 (Altered), 0 (Not Altered) | Altered, Not Altered |
| Pathway Alteration Data (Derived) | p53, PI3K, Cell.cycle, RTK.RAS, WNT, TGF.Beta, Hippo | Alteration status for specific biological pathways. | Binary | 1 (Altered), 0 (Not Altered) | Altered, Not Altered |
| **Outcome Variables** | | | | | |
| Cancer diagnosis dataset | os_dx_status | Overall Survival from Diagnosis: Status indicator (1 = Dead, 0 = Censored). | Numeric | 1, 0 | No recategorization |
| Cancer diagnosis dataset | tt_os_dx_days | Overall Survival from Diagnosis: Time in days from cancer diagnosis to death or last follow-up. | Numeric | Continuous (Days) | No recategorization |
| Cancer diagnosis dataset | pfs_i_or_m_adv_status | Progression-Free Survival (Imaging or Medical Oncologist Assessment) from Diagnosis: Status indicator (1 = Progression or Death, 0 = Censored). | Numeric | 1, 0 | No recategorization |
| Cancer diagnosis dataset | tt_pfs_i_or_m_adv_days | Progression-Free Survival (Imaging or Medical Oncologist Assessment) from Diagnosis: Time in days from cancer diagnosis to progression/death or last follow-up. | Numeric | Continuous (Days) | No recategorization |
| Cancer-directed regimen dataset | os_g_status | Overall Survival from Start of Cancer-Directed Regimen: Status indicator (1 = Dead, 0 = Censored). | Numeric | 1, 0 | No recategorization |
| Cancer-directed regimen dataset | tt_os_g_days | Overall Survival from Start of Cancer-Directed Regimen: Time in days from regimen start to death or last follow-up. | Numeric | Continuous (Days) | No recategorization |
| Cancer-directed regimen dataset | pfs_i_or_m_g_status | Progression-Free Survival (Imaging or Medical Oncologist Assessment) from Start of Cancer-Directed Regimen: Status indicator (1 = Progression or Death, 0 = Censored). | Numeric | 1, 0 | No recategorization |
| Cancer-directed regimen dataset | tt_pfs_i_or_m_g_days | Progression-Free Survival (Imaging or Medical Oncologist Assessment) from Start of Cancer-Directed Regimen: Time in days from regimen start to progression/death or last follow-up. | Numeric | Continuous (Days) | No recategorization |
| Derived | reg_to_dmets_days | Distant Recurrence-Free Survival from Treatment (Stage I-III): Time in days from first regimen start to distant metastasis or last follow-up. Derived as dx_to_dmets_days - regimen_start_date. | Derived Numeric | Continuous (Days) | No recategorization |

**Supplemental Table 2.** Mapping of drug names to their respective classifications for the definition of treatment categories.

| **Drug Name** | **Category** |
| --- | --- |
| Cyclophosphamide | **Chemotherapy** |
| Paclitaxel |  |
| Doxorubicin Hydrochloride |  |
| Capecitabine |  |
| Carboplatin |  |
| Eribulin Mesylate |  |
| Gemcitabine Hydrochloride |  |
| Docetaxel |  |
| Vinorelbine Tartrate |  |
| Nabpaclitaxel |  |
| Fluorouracil |  |
| Methotrexate |  |
| Cisplatin |  |
| Epirubicin Hydrochloride |  |
| Ifosfamide |  |
| Pegylated Liposomal Doxorubicin Hydrochloride |  |
| Paclitaxel Loaded Polymeric Micelle |  |
| Ixabepilone |  |
| Irinotecan Hydrochloride |  |
| Cabozantinib Smalate |  |
| Etoposide |  |
| Other antineoplastic |  |
| Leucovorin Calcium |  |
| Dacarbazine |  |
| Oxaliplatin |  |
| Temozolomide |  |
| Anthracycline |  |
| Bendamustine Hydrochloride |  |
| High dose chemotherapy |  |
| Pemetrexed Disodium |  |
| Topotecan Hydrochloride |  |
| Vincristine Sulfate |  |
| Tamoxifen Citrate | **Hormone Therapy** |
| Letrozole |  |
| Fulvestrant |  |
| Leuprolide Acetate |  |
| Anastrozole |  |
| Exemestane |  |
| Goserlin Acetate |  |
| Enzalutamide |  |
| Toremifene |  |
| Aromatase Inhibitors |  |
| Aromatase inhibitor |  |
| Bicalutamide |  |
| Megestrol Acetate |  |
| Other hormone |  |
| Other aromatase inhibitor |  |
| Pembrolizumab | **Immunotherapy** |
| Nivolumab |  |
| Atezolizumab |  |
| Ipilimumab |  |
| Avelumab |  |
| Trastuzumab | **HER2 therapy** |
| Pertuzumab |  |
| Neratinib |  |
| Trastuzumab Emtansine |  |
| Lapatinib Ditosylate |  |
| Trastuzumab Deruxtecan |  |
| Pertuzumab-Trastuzumab-Hyaluronidase-ZZXF |  |
| Trastuzumab/Hyaluronidase-oysk |  |
| Tucatinib |  |
| Ribociclib | **CDK4/6** |
| Palbociclib |  |
| Abemaciclib |  |
| Everolimus | **PI3K/mTOR inhibitors** |
| Alpelisib |  |
| Bevacizumab | **Other** |
| SACITUZUMAB GOVITECAN-HZIY |  |
| sacituzumab govitecan |  |
| Sacituzumab Govitecan |  |
| Sorafenib Tosylate |  |
| Olaparib |  |
| Olaratumab |  |
| Pazopanib Hydrochloride |  |
| Lenvatinib Mesylate |  |
| Yttrium Y90 Ibritumomab Tiuxetan |  |
| AARSOTA |  |
| Apatinib |  |
| BCG Solution |  |
| Cetuximab |  |
| Dasatinib |  |
| Erlotinib Hydrochloride |  |
| Floxuridine |  |
| Interleukin-2 |  |
| Irinotecan liposome |  |
| LHRH |  |
| Larotrectinib |  |
| Lurbinectedin |  |
| Metformin |  |
| Pioglitazone |  |
| Ponatinib Hydrochloride |  |
| Ramucirumab |  |
| Rituximab |  |
| TACE |  |
| Talazoparib |  |
| Taselisib |  |
| Tegafurgimeraciloteracil Potassium |  |
| Trametinib |  |
| Tremelimumab |  |
| Unknown |  |
| unknown |  |

**Supplemental Table 3.** Treatment and Clinical Procedures overall and by *PIK3CA* Status on HER2+ patients.

| **Characteristic** | **Overall** | **Not Altered** | **Altered** | **p-value***^2^* |
| --- | --- | --- | --- | --- |
|  | **N = 212^1^** | **N = 154^1^** | **N = 58^1^** |  |
| **Days from Cancer to First Oncologist Visit** | 31 (14, 282) | 28 (14, 260) | 40 (18, 282) | 0.3 |
| Missing | 1 | 1 | 0 |  |
| **Total Pathology Reports** | 6 (4, 8) | 6 (4, 7) | 7 (4, 9) | 0.089 |
| **CA15-3 Result** | 21 (12, 37) | 19 (12, 29) | 24 (15, 51) | 0.07 |
| Missing | 117 | 88 | 29 |  |
| **CA27-29 Result** | 27 (18, 53) | 30 (18, 53) | 26 (20, 49) | 0.7 |
| Missing | 145 | 107 | 38 |  |
| **Had CT Scan** |  |  |  | **0.022** |
| Yes | 177 (83%) | 123 (80%) | 54 (93%) |  |
| No | 35 (17%) | 31 (20%) | 4 (6.9%) |  |
| **Had MRI** |  |  |  | 0.8 |
| Yes | 187 (88%) | 135 (88%) | 52 (90%) |  |
| No | 25 (12%) | 19 (12%) | 6 (10%) |  |
| **Had PET or PET-CT Scan** |  |  |  | 0.2 |
| Yes | 141 (67%) | 98 (64%) | 43 (74%) |  |
| No | 71 (33%) | 56 (36%) | 15 (26%) |  |
| **Had Bone Scan** |  |  |  | **0.013** |
| Yes | 116 (55%) | 76 (49%) | 40 (69%) |  |
| No | 96 (45%) | 78 (51%) | 18 (31%) |  |
| **Had Mammogram** |  |  |  | 0.7 |
| Yes | 152 (72%) | 109 (71%) | 43 (74%) |  |
| No | 60 (28%) | 45 (29%) | 15 (26%) |  |
| **Days from Cancer to First Reference Imaging** | 7 (-6, 614) | 7 (-8, 441) | 42 (-5, 856) | 0.3 |
| Missing | 15 | 11 | 4 |  |
| **Metastasis to Abdomen** |  |  |  | 0.5 |
| Yes | 79 (37%) | 55 (36%) | 24 (41%) |  |
| No | 133 (63%) | 99 (64%) | 34 (59%) |  |
| **Metastasis to Bone** |  |  |  | 0.089 |
| Yes | 114 (54%) | 77 (50%) | 37 (64%) |  |
| No | 98 (46%) | 77 (50%) | 21 (36%) |  |
| **Metastasis to Brain** |  |  |  | 0.3 |
| Yes | 89 (42%) | 61 (40%) | 28 (48%) |  |
| No | 123 (58%) | 93 (60%) | 30 (52%) |  |
| **Metastasis to Extremity** |  |  |  | >0.9 |
| Yes | 6 (2.8%) | 5 (3.2%) | 1 (1.7%) |  |
| No | 206 (97%) | 149 (97%) | 57 (98%) |  |
| **Metastasis to Head & Neck** |  |  |  | 0.11 |
| Yes | 39 (18%) | 24 (16%) | 15 (26%) |  |
| No | 173 (82%) | 130 (84%) | 43 (74%) |  |
| **Hematologic Metastasis** |  |  |  | >0.9 |
| Yes | 14 (6.6%) | 10 (6.5%) | 4 (6.9%) |  |
| No | 198 (93%) | 144 (94%) | 54 (93%) |  |
| **Metastasis to Liver** |  |  |  | 0.4 |
| Yes | 105 (50%) | 73 (47%) | 32 (55%) |  |
| No | 107 (50%) | 81 (53%) | 26 (45%) |  |
| **Metastasis to Pelvis** |  |  |  | 0.1 |
| Yes | 35 (17%) | 21 (14%) | 14 (24%) |  |
| No | 177 (83%) | 133 (86%) | 44 (76%) |  |
| **Metastasis to Thorax** |  |  |  | 0.8 |
| Yes | 125 (59%) | 92 (60%) | 33 (57%) |  |
| No | 87 (41%) | 62 (40%) | 25 (43%) |  |
| **Metastasis to Trunk** |  |  |  | >0.9 |
| Yes | 1 (0.5%) | 1 (0.6%) | 0 (0%) |  |
| No | 211 (100%) | 153 (99%) | 58 (100%) |  |
| **Months from Diagnosis to Distant Metastasis*** | 26 (12, 57) | 22 (10, 43) | 38 (23, 73) | **0.018** |
| Missing | 97 | 73 | 24 |  |
| **Neoadjuvant Tx Before Pathologic Stage** |  |  |  | 0.8 |
| Unknown/Not Applicable | 13 (6.1%) | 10 (6.5%) | 3 (5.2%) |  |
| No | 108 (51%) | 76 (49%) | 32 (55%) |  |
| Yes | 91 (43%) | 68 (44%) | 23 (40%) |  |
| **Received Chemotherapy** |  |  |  | 0.13 |
| Yes | 207 (98%) | 152 (99%) | 55 (95%) |  |
| No | 5 (2.4%) | 2 (1.3%) | 3 (5.2%) |  |
| **Received Hormone Therapy** |  |  |  | **0.05** |
| Yes | 142 (67%) | 97 (63%) | 45 (78%) |  |
| No | 70 (33%) | 57 (37%) | 13 (22%) |  |
| **Received Immunotherapy** |  |  |  | >0.9 |
| Yes | 19 (9.0%) | 14 (9.1%) | 5 (8.6%) |  |
| No | 193 (91%) | 140 (91%) | 53 (91%) |  |
| **Received HER2 Therapy** |  |  |  | **0.049** |
| Yes | 194 (92%) | 145 (94%) | 49 (84%) |  |
| No | 18 (8.5%) | 9 (5.8%) | 9 (16%) |  |
| **Received CDK4/6 Inhibitors** |  |  |  | 0.076 |
| Yes | 29 (14%) | 17 (11%) | 12 (21%) |  |
| No | 183 (86%) | 137 (89%) | 46 (79%) |  |
| **Received PI3K/mTOR Inhibitors** |  |  |  | 0.2 |
| Yes | 19 (9.0%) | 11 (7.1%) | 8 (14%) |  |
| No | 193 (91%) | 143 (93%) | 50 (86%) |  |
| **Received Other Therapy** |  |  |  | 0.4 |
| Yes | 26 (12%) | 17 (11%) | 9 (16%) |  |
| No | 186 (88%) | 137 (89%) | 49 (84%) |  |
| **Regimen Number** |  |  |  | 0.14 |
| 1 | 203 (97%) | 149 (98%) | 54 (95%) |  |
| 2 | 2 (1.0%) | 1 (0.7%) | 1 (1.8%) |  |
| 3 | 2 (1.0%) | 0 (0%) | 2 (3.5%) |  |
| 4 | 2 (1.0%) | 2 (1.3%) | 0 (0%) |  |
| Missing | 3 | 2 | 1 |  |
| **Number of Drugs in Regimen** |  |  |  | 0.6 |
| 1 | 17 (8.1%) | 11 (7.2%) | 6 (11%) |  |
| 2 | 37 (18%) | 27 (18%) | 10 (18%) |  |
| 3 | 88 (42%) | 62 (41%) | 26 (46%) |  |
| 4 | 43 (21%) | 35 (23%) | 8 (14%) |  |
| 5 | 24 (11%) | 17 (11%) | 7 (12%) |  |
| Missing | 3 | 2 | 1 |  |
| **Regimen Part of Clinical Trial** |  |  |  | 0.4 |
| No | 195 (92%) | 144 (94%) | 51 (88%) |  |
| Yes | 14 (6.6%) | 8 (5.2%) | 6 (10%) |  |
| Unknown | 3 (1.4%) | 2 (1.3%) | 1 (1.7%) |  |
| **Months from Diagnosis to Regimen Start** | 1 (1, 2) | 1 (1, 2) | 1 (1, 2) | 0.4 |
| Missing | 3 | 2 | 1 |  |
| **Days from Regimen Start to End** | 152 (99, 273) | 153 (101, 260) | 144 (93, 310) | >0.9 |
| Missing | 9 | 5 | 4 |  |
| *^1^*Median (Q1, Q3); n (%) | | | | |
| *^2^*Kruskal-Wallis rank sum test; Fisher's exact test  **Early-stage patients* | | | | |

**Supplemental Table 4.** Frequency of Specific Drugs Overall and by *PIK3CA* Status

| **Drug** | ***PIK3CA* not mutated** | ***PIK3CA*m** | **p-value** |
| --- | --- | --- | --- |
| **Chemotherapy** |  |  |  |
| Paclitaxel | 114 (74%) | 43 (74.1%) | 0.524 |
| Cyclophosphamide | 86 (55.8%) | 29 (50%) | 0.293 |
| Doxorubicin Hydrochloride | 80 (51.9%) | 24 (41.4%) | 0.15 |
| Capecitabine | 64 (41.6%) | 30 (51.7%) | 0.644 |
| Docetaxel | 58 (37.7%) | 16 (27.6%) | 0.152 |
| Carboplatin | 46 (29.9%) | 25 (43.1%) | 0.293 |
| Vinorelbine Tartrate | 35 (22.7%) | 15 (25.9%) | >0.999 |
| Eribulin Mesylate | 24 (15.6%) | 14 (24.1%) | 0.37 |
| Gemcitabine Hydrochloride | 22 (14.3%) | 12 (20.7%) | 0.455 |
| Nabpaclitaxel | 21 (13.6%) | 6 (10.3%) | 0.525 |
| Pegylated Liposomal Doxorubicin Hydrochloride | 16 (10.4%) | 9 (15.5%) | 0.511 |
| Fluorouracil | 7 (4.5%) | 4 (6.9%) | 0.742 |
| Methotrexate | 6 (3.9%) | 2 (3.4%) | >0.999 |
| Cabozantinib Smalate | 4 (2.6%) | 2 (3.4%) | >0.999 |
| Epirubicin Hydrochloride | 3 (1.9%) | 2 (3.4%) | 0.637 |
| Irinotecan Hydrochloride | 2 (1.3%) | 2 (3.4%) | 0.587 |
| Ixabepilone | 2 (1.3%) | 1 (1.7%) | >0.999 |
| Cisplatin | 1 (0.6%) | 0 (0%) | >0.999 |
| Etoposide | 0 (0%) | 1 (1.7%) | 0.297 |
| Leucovorin Calcium | 1 (0.6%) | 0 (0%) | >0.999 |
| Other antineoplastic | 0 (0%) | 1 (1.7%) | 0.297 |
| Oxaliplatin | 1 (0.6%) | 0 (0%) | >0.999 |
| **Hormone Therapy** |  |  |  |
| Tamoxifen Citrate | 60 (39%) | 34 (58.6%) | 0.165 |
| Letrozole | 44 (28.6%) | 22 (37.9%) | 0.496 |
| Leuprolide Acetate | 39 (25.3%) | 21 (36.2%) | 0.389 |
| Fulvestrant | 28 (18.2%) | 16 (27.6%) | 0.321 |
| Anastrozole | 30 (19.5%) | 12 (20.7%) | >0.999 |
| Exemestane | 20 (13%) | 14 (24.1%) | 0.183 |
| Goserlin Acetate | 8 (5.2%) | 6 (10.3%) | 0.377 |
| Enzalutamide | 2 (1.3%) | 0 (0%) | >0.999 |
| Bicalutamide | 1 (0.6%) | 0 (0%) | >0.999 |
| **Immunotherapy** |  |  |  |
| Pembrolizumab | 11 (7.1%) | 5 (8.6%) | >0.999 |
| Atezolizumab | 2 (1.3%) | 0 (0%) | >0.999 |
| Ipilimumab | 1 (0.6%) | 0 (0%) | >0.999 |
| Nivolumab | 1 (0.6%) | 0 (0%) | >0.999 |
| **HER2 Therapy** | | | |
| Trastuzumab | 145 (94.2%) | 49 (84.5%) | 0.158 |
| Pertuzumab | 113 (73.4%) | 38 (65.5%) | 0.227 |
| Trastuzumab Emtansine | 61 (39.6%) | 30 (51.7%) | 0.482 |
| Lapatinib Ditosylate | 45 (29.2%) | 19 (32.8%) | >0.999 |
| Neratinib | 8 (5.2%) | 1 (1.7%) | 0.295 |
| Trastuzumab Deruxtecan | 5 (3.2%) | 1 (1.7%) | 0.676 |
| Trastuzumab/Hyaluronidase-oysk | 2 (1.3%) | 1 (1.7%) | >0.999 |
| Tucatinib | 2 (1.3%) | 0 (0%) | >0.999 |
| Pertuzumab-Trastuzumab-Hyaluronidase-ZZXF | 1 (0.6%) | 0 (0%) | >0.999 |
| **CDK4/6 Inhibitors** | | | |
| Palbociclib | 16 (10.4%) | 11 (19%) | 0.209 |
| Abemaciclib | 1 (0.6%) | 2 (3.4%) | 0.213 |
| Ribociclib | 1 (0.6%) | 0 (0%) | >0.999 |
| **PI3K/mTOR Inhibitors** |  |  |  |
| Alpelisib | 0 | 0 | - |
| Everolimus | 11 (7.1%) | 8 (13.8%) | 0.311 |
| **Other** | | | |
| Bevacizumab | 12 (7.8%) | 6 (10.3%) | 0.796 |
| Olaparib | 3 (1.9%) | 1 (1.7%) | >0.999 |
| AARSOTA | 0 (0%) | 1 (1.7%) | 0.297 |
| Apatinib | 1 (0.6%) | 0 (0%) | >0.999 |
| Dasatinib | 1 (0.6%) | 0 (0%) | >0.999 |
| Pazopanib Hydrochloride | 1 (0.6%) | 0 (0%) | >0.999 |
| Ramucirumab | 1 (0.6%) | 0 (0%) | >0.999 |
| Sorafenib Tosylate | 1 (0.6%) | 0 (0%) | >0.999 |
| sacituzumab govitecan | 0 (0%) | 1 (1.7%) | 0.297 |
| unknown | 0 (0%) | 1 (1.7%) | 0.297 |

**Supplemental Table 5.** Co-occurring Gene Alterations Overall and by *PIK3CA* Status.

| **Characteristic** | **Overall** | **Not Altered** | **Altered** | **p-value***^2^* |
| --- | --- | --- | --- | --- |
|  | **N = 212^1^** | **N = 154^1^** | **N = 58^1^** |  |
| **ARID1A** |  |  |  | >0.9 |
| Altered | 19 (9.0%) | 14 (9.1%) | 5 (8.6%) |  |
| Not Altered | 193 (91%) | 140 (91%) | 53 (91%) |  |
| **ARID1B** |  |  |  | 0.5 |
| Altered | 13 (6.1%) | 11 (7.1%) | 2 (3.4%) |  |
| Not Altered | 199 (94%) | 143 (93%) | 56 (97%) |  |
| **ATM** |  |  |  | >0.9 |
| Altered | 11 (5.2%) | 8 (5.2%) | 3 (5.2%) |  |
| Not Altered | 201 (95%) | 146 (95%) | 55 (95%) |  |
| **BRCA2** |  |  |  | 0.12 |
| Altered | 13 (6.1%) | 12 (7.8%) | 1 (1.7%) |  |
| Not Altered | 199 (94%) | 142 (92%) | 57 (98%) |  |
| **BRIP1** |  |  |  | >0.9 |
| Altered | 24 (11%) | 18 (12%) | 6 (10%) |  |
| Not Altered | 188 (89%) | 136 (88%) | 52 (90%) |  |
| **CCND1** |  |  |  | 0.5 |
| Altered | 34 (16%) | 23 (15%) | 11 (19%) |  |
| Not Altered | 178 (84%) | 131 (85%) | 47 (81%) |  |
| **CD79B** |  |  |  | 0.8 |
| Altered | 16 (7.5%) | 11 (7.1%) | 5 (8.6%) |  |
| Not Altered | 196 (92%) | 143 (93%) | 53 (91%) |  |
| **CDH1** |  |  |  | 0.5 |
| Altered | 13 (6.1%) | 11 (7.1%) | 2 (3.4%) |  |
| Not Altered | 199 (94%) | 143 (93%) | 56 (97%) |  |
| **CDK12** |  |  |  | 0.5 |
| Altered | 95 (45%) | 67 (44%) | 28 (48%) |  |
| Not Altered | 117 (55%) | 87 (56%) | 30 (52%) |  |
| **ERBB2** |  |  |  | 0.2 |
| Altered | 160 (75%) | 120 (78%) | 40 (69%) |  |
| Not Altered | 52 (25%) | 34 (22%) | 18 (31%) |  |
| **ESR1** |  |  |  | 0.5 |
| Altered | 11 (5.2%) | 7 (4.5%) | 4 (6.9%) |  |
| Not Altered | 201 (95%) | 147 (95%) | 54 (93%) |  |
| **FANCA** |  |  |  | >0.9 |
| Altered | 11 (5.2%) | 8 (5.2%) | 3 (5.2%) |  |
| Not Altered | 201 (95%) | 146 (95%) | 55 (95%) |  |
| **FGF19** |  |  |  | 0.4 |
| Altered | 20 (9.4%) | 13 (8.4%) | 7 (12%) |  |
| Not Altered | 192 (91%) | 141 (92%) | 51 (88%) |  |
| **FGF3** |  |  |  | 0.4 |
| Altered | 19 (9.0%) | 12 (7.8%) | 7 (12%) |  |
| Not Altered | 193 (91%) | 142 (92%) | 51 (88%) |  |
| **FGF4** |  |  |  | 0.3 |
| Altered | 21 (9.9%) | 13 (8.4%) | 8 (14%) |  |
| Not Altered | 191 (90%) | 141 (92%) | 50 (86%) |  |
| **FGFR1** |  |  |  | 0.6 |
| Altered | 23 (11%) | 18 (12%) | 5 (8.6%) |  |
| Not Altered | 189 (89%) | 136 (88%) | 53 (91%) |  |
| **GATA3** |  |  |  | 0.5 |
| Altered | 26 (12%) | 21 (14%) | 5 (8.6%) |  |
| Not Altered | 186 (88%) | 133 (86%) | 53 (91%) |  |
| **GNAS** |  |  |  | 0.5 |
| Altered | 11 (5.2%) | 7 (4.5%) | 4 (6.9%) |  |
| Not Altered | 201 (95%) | 147 (95%) | 54 (93%) |  |
| **HOXB13** |  |  |  | 0.7 |
| Altered | 11 (5.2%) | 9 (5.8%) | 2 (3.4%) |  |
| Not Altered | 201 (95%) | 145 (94%) | 56 (97%) |  |
| **IKZF3** |  |  |  | 0.4 |
| Altered | 34 (16%) | 27 (18%) | 7 (12%) |  |
| Not Altered | 178 (84%) | 127 (82%) | 51 (88%) |  |
| **KMT2D** |  |  |  | 0.5 |
| Altered | 13 (6.1%) | 11 (7.1%) | 2 (3.4%) |  |
| Not Altered | 199 (94%) | 143 (93%) | 56 (97%) |  |
| **MDM2** |  |  |  | >0.9 |
| Altered | 11 (5.2%) | 8 (5.2%) | 3 (5.2%) |  |
| Not Altered | 201 (95%) | 146 (95%) | 55 (95%) |  |
| **MYC** |  |  |  | 0.8 |
| Altered | 33 (16%) | 25 (16%) | 8 (14%) |  |
| Not Altered | 179 (84%) | 129 (84%) | 50 (86%) |  |
| **NBN** |  |  |  | 0.8 |
| Altered | 14 (6.6%) | 11 (7.1%) | 3 (5.2%) |  |
| Not Altered | 198 (93%) | 143 (93%) | 55 (95%) |  |
| **NF1** |  |  |  | 0.8 |
| Altered | 22 (10%) | 17 (11%) | 5 (8.6%) |  |
| Not Altered | 190 (90%) | 137 (89%) | 53 (91%) |  |
| **PPM1D** |  |  |  | >0.9 |
| Altered | 17 (8.0%) | 13 (8.4%) | 4 (6.9%) |  |
| Not Altered | 195 (92%) | 141 (92%) | 54 (93%) |  |
| **PRKAR1A** |  |  |  | 0.3 |
| Altered | 13 (6.1%) | 8 (5.2%) | 5 (8.6%) |  |
| Not Altered | 199 (94%) | 146 (95%) | 53 (91%) |  |
| **RAD21** |  |  |  | >0.9 |
| Altered | 19 (9.0%) | 14 (9.1%) | 5 (8.6%) |  |
| Not Altered | 193 (91%) | 140 (91%) | 53 (91%) |  |
| **RAD51C** |  |  |  | 0.4 |
| Altered | 16 (7.5%) | 10 (6.5%) | 6 (10%) |  |
| Not Altered | 196 (92%) | 144 (94%) | 52 (90%) |  |
| **RARA** |  |  |  | >0.9 |
| Altered | 34 (16%) | 25 (16%) | 9 (16%) |  |
| Not Altered | 178 (84%) | 129 (84%) | 49 (84%) |  |
| **RECQL4** |  |  |  | 0.076 |
| Altered | 16 (7.5%) | 15 (9.7%) | 1 (1.7%) |  |
| Not Altered | 196 (92%) | 139 (90%) | 57 (98%) |  |
| **RNF43** |  |  |  | >0.9 |
| Altered | 15 (7.1%) | 11 (7.1%) | 4 (6.9%) |  |
| Not Altered | 197 (93%) | 143 (93%) | 54 (93%) |  |
| **ROS1** |  |  |  | 0.2 |
| Altered | 14 (6.6%) | 8 (5.2%) | 6 (10%) |  |
| Not Altered | 198 (93%) | 146 (95%) | 52 (90%) |  |
| **SOX9** |  |  |  | >0.9 |
| Altered | 11 (5.2%) | 8 (5.2%) | 3 (5.2%) |  |
| Not Altered | 201 (95%) | 146 (95%) | 55 (95%) |  |
| **SPOP** |  |  |  | >0.9 |
| Altered | 26 (12%) | 19 (12%) | 7 (12%) |  |
| Not Altered | 186 (88%) | 135 (88%) | 51 (88%) |  |
| **TP53** |  |  |  | 0.5 |
| Altered | 142 (67%) | 101 (66%) | 41 (71%) |  |
| Not Altered | 70 (33%) | 53 (34%) | 17 (29%) |  |
| *^1^*n (%) | | | | |
| *^2^*Fisher's exact test | | | | |

**Supplemental Table 6.** Co-occurring Pathway Alterations Overall and by *PIK3CA* Status.

| **Characteristic** | **Overall** | **Not Altered** | **Altered** | **p-value***^2^* |
| --- | --- | --- | --- | --- |
|  | **N = 212^1^** | **N = 154^1^** | **N = 58^1^** |  |
| **RTK.RAS** |  |  |  | 0.6 |
| Altered | 191 (90%) | 140 (91%) | 51 (88%) |  |
| Not Altered | 21 (9.9%) | 14 (9.1%) | 7 (12%) |  |
| **Cell.cycle** |  |  |  | 0.5 |
| Altered | 54 (25%) | 37 (24%) | 17 (29%) |  |
| Not Altered | 158 (75%) | 117 (76%) | 41 (71%) |  |
| **p53** |  |  |  | 0.6 |
| Altered | 162 (76%) | 116 (75%) | 46 (79%) |  |
| Not Altered | 50 (24%) | 38 (25%) | 12 (21%) |  |
| **PI3K** |  |  |  | **<0.001** |
| Altered | 90 (42%) | 32 (21%) | 58 (100%) |  |
| Not Altered | 122 (58%) | 122 (79%) | 0 (0%) |  |
| **Hippo** |  |  |  | **0.022** |
| Altered | 13 (6.1%) | 13 (8.4%) | 0 (0%) |  |
| Not Altered | 199 (94%) | 141 (92%) | 58 (100%) |  |
| *^1^*n (%) | | | | |
| *^2^*Fisher's exact test | | | | |

**Supplemental Table 7.** Univariable and multivariable Cox Proportional Hazard Models for Overall Survival after diagnosis for HER2+/ER+ patients.

|  |  | **Univariable** | | | **Multivariable** | | |
| --- | --- | --- | --- | --- | --- | --- | --- |
| **Characteristic** | **N** | **HR** | **95% CI** | **p-value** | **aHR** | **95% CI** | **p-value** |
| Age at Diagnosis | 132 | 1.1 | 1.00, 1.10 | **0.031** |  |  |  |
| Race | 132 |  |  |  |  |  |  |
| White |  | — | — |  |  |  |  |
| Black |  | 0.9 | 0.21, 3.67 | 0.9 |  |  |  |
| Asian |  | 0.6 | 0.15, 2.57 | 0.5 |  |  |  |
| Other/Unknown |  | 0.7 | 0.09, 4.75 | 0.7 |  |  |  |
| Ethnicity | 132 |  |  |  |  |  |  |
| Non-Hispanic |  | — | — |  |  |  |  |
| Hispanic |  | 1.4 | 0.34, 6.01 | 0.6 |  |  |  |
| Unknown |  | 0 | 0.00, Inf | >0.9 |  |  |  |
| Tumor Laterality | 132 |  |  |  |  |  |  |
| Left |  | — | — |  |  |  |  |
| Other/Unknown |  | 1.2 | 0.51, 2.72 | 0.7 |  |  |  |
| Right |  | 0.6 | 0.32, 1.19 | 0.15 |  |  |  |
| Histology | 132 |  |  |  |  |  |  |
| Invasive ductal carcinoma |  | — | — |  |  |  |  |
| Invasive lobular carcinoma |  | 3 | 0.72, 12.5 | 0.13 |  |  |  |
| Other histology |  | 4.4 | 0.59, 33.0 | 0.15 |  |  |  |
| PR Status | 132 |  |  |  |  |  |  |
| Positive |  | — | — |  |  |  |  |
| Negative |  | 1.5 | 0.66, 3.32 | 0.3 |  |  |  |
| Unknown/Not Tested |  | 0 | 0.00, Inf | >0.9 |  |  |  |
| Stage at Diagnosis | 132 |  |  |  |  |  |  |
| Early Stage (I-III) |  | — | — |  |  |  |  |
| Stage IV |  | 1.5 | 0.75, 2.98 | 0.3 |  |  |  |
| Unknown/Other Stage |  |  |  |  |  |  |  |
| Received Chemotherapy | 132 |  |  |  |  |  |  |
| Yes |  | — | — |  |  |  |  |
| No |  | 0 | 0.00, Inf | >0.9 |  |  |  |
| Received Hormone Therapy | 132 |  |  |  |  |  |  |
| Yes |  | — | — |  |  |  |  |
| No |  | 2.1 | 0.50, 9.05 | 0.3 |  |  |  |
| Received Immunotherapy | 132 |  |  |  |  |  |  |
| Yes |  | — | — |  |  |  |  |
| No |  | 0.6 | 0.23, 1.50 | 0.3 |  |  |  |
| Received HER2 Therapy | 132 |  |  |  |  |  |  |
| Yes |  | — | — |  |  |  |  |
| No |  | 0.5 | 0.22, 1.15 | 0.1 |  |  |  |
| Received CDK4/6 Inhibitors | 132 |  |  |  |  |  |  |
| Yes |  | — | — |  |  |  |  |
| No |  | 1.1 | 0.58, 2.05 | 0.8 |  |  |  |
| Received PI3K Inhibitors | 132 |  |  |  |  |  |  |
| Yes |  | — | — |  |  |  |  |
| No |  | 1 | 0.50, 2.04 | >0.9 |  |  |  |
| Received Other Therapy | 132 |  |  |  |  |  |  |
| Yes |  | — | — |  |  |  |  |
| No |  | 2.2 | 0.52, 8.94 | 0.3 |  |  |  |
| TP53 Alteration | 132 |  |  |  |  |  |  |
| Altered |  | — | — |  |  |  |  |
| Not Altered |  | 0.7 | 0.41, 1.34 | 0.3 |  |  |  |
| *PIK3CA* Alteration | 132 | 1.2 | 0.66, 2.08 | 0.6 |  |  |  |
| ERBB2 Alteration | 132 |  |  |  |  |  |  |
| Altered |  | — | — |  |  |  |  |
| Not Altered |  | 1.3 | 0.73, 2.33 | 0.4 |  |  |  |
| p53 Pathway | 132 |  |  |  |  |  |  |
| Altered |  | — | — |  |  |  |  |
| Not Altered |  | 0.9 | 0.48, 1.63 | 0.7 |  |  |  |
| PI3K Pathway | 132 |  |  |  |  |  |  |
| Altered |  | — | — |  |  |  |  |
| Not Altered |  | 1.1 | 0.64, 2.00 | 0.7 |  |  |  |
| Cell Cycle Pathway | 132 |  |  |  |  |  |  |
| Altered |  | — | — |  |  |  |  |
| Not Altered |  | 1 | 0.53, 1.87 | >0.9 |  |  |  |
| Abbreviations: CI = Confidence Interval, HR = Hazard Ratio, aHR = adjusted Hazard Ratio | | | | | | | |

**Supplemental Table 8.** Univariable and multivariable Cox Proportional Hazard Models for Overall Survival after diagnosis for HER2+/ER- patients.

|  |  | **Univariable** | | | **Multivariable** | | |
| --- | --- | --- | --- | --- | --- | --- | --- |
| **Characteristic** | **N** | **HR** | **95% CI** | **p-value** | **aHR** | **95% CI** | **p-value** |
| Age at Diagnosis | 80 | 1 | 0.96, 1.07 | 0.6 |  |  |  |
| Race | 80 |  |  |  |  |  |  |
| White |  | — | — |  |  |  |  |
| Black |  | 1.1 | 0.47, 2.51 | 0.9 |  |  |  |
| Asian |  | 1 | 0.13, 7.36 | >0.9 |  |  |  |
| Other/Unknown |  | 0.5 | 0.16, 1.77 | 0.3 |  |  |  |
| Ethnicity | 80 |  |  |  |  |  |  |
| Non-Hispanic |  | — | — |  | — | — |  |
| Hispanic |  | 0 | 0.00, Inf | >0.9 | 0 | 0.00, Inf | >0.9 |
| Unknown |  | 1.8 | 0.25, 13.6 | 0.6 | 2.05 | 0.27, 15.7 | 0.5 |
| Tumor Laterality | 80 |  |  |  |  |  |  |
| Left |  | — | — |  |  |  |  |
| Other/Unknown |  | 1.5 | 0.66, 3.51 | 0.3 |  |  |  |
| Right |  | 1.2 | 0.54, 2.50 | 0.7 |  |  |  |
| Histology | 80 |  |  |  |  |  |  |
| Invasive ductal carcinoma |  | — | — |  |  |  |  |
| Invasive lobular carcinoma |  | 2 | 0.26, 14.9 | 0.5 |  |  |  |
| Other histology |  | 5.8 | 0.75, 44.5 | 0.093 |  |  |  |
| PR Status | 80 |  |  |  |  |  |  |
| Positive |  | — | — |  |  |  |  |
| Negative |  | 3.5 | 0.47, 25.5 | 0.2 |  |  |  |
| Unknown/Not Tested |  |  |  |  |  |  |  |
| Stage at Diagnosis | 80 |  |  |  |  |  |  |
| Early Stage (I-III) |  | — | — |  |  |  |  |
| Stage IV |  | 0.7 | 0.35, 1.47 | 0.4 |  |  |  |
| Unknown/Other Stage |  |  |  |  |  |  |  |
| Breast Cancer Subtype | 80 |  |  |  |  |  |  |
| HER2+, HR- |  | — | — |  |  |  |  |
| HER2+, HR+ |  | 0.3 | 0.04, 2.11 | 0.2 |  |  |  |
| Received Chemotherapy | 80 |  |  |  |  |  |  |
| Yes |  | — | — |  |  |  |  |
| No |  | 1.8 | 0.43, 7.54 | 0.4 |  |  |  |
| Received Hormone Therapy | 80 |  |  |  |  |  |  |
| Yes |  | — | — |  | — | — |  |
| No |  | 2.2 | 1.02, 4.70 | **0.045** | 2.74 | 1.24, 6.08 | **0.013** |
| Received Immunotherapy | 80 |  |  |  |  |  |  |
| Yes |  | — | — |  | — | — |  |
| No |  | 0.4 | 0.16, 0.82 | **0.015** | 0.41 | 0.17, 0.98 | **0.046** |
| Received HER2 Therapy | 80 |  |  |  |  |  |  |
| Yes |  | — | — |  | — | — |  |
| No |  | 4.4 | 1.02, 18.8 | **0.047** | 5.06 | 1.12, 22.8 | **0.035** |
| Received CDK4/6 Inhibitors | 80 |  |  |  |  |  |  |
| Yes |  | — | — |  |  |  |  |
| No |  | 1.1 | 0.26, 4.65 | 0.9 |  |  |  |
| Received PI3K Inhibitors | 80 |  |  |  |  |  |  |
| Yes |  | — | — |  |  |  |  |
| No |  | 1.1 | 0.15, 8.12 | >0.9 |  |  |  |
| Received Other Therapy | 80 |  |  |  |  |  |  |
| Yes |  | — | — |  |  |  |  |
| No |  | 2.6 | 0.33, 20.8 | 0.4 |  |  |  |
| TP53 Alteration | 80 |  |  |  |  |  |  |
| Altered |  | — | — |  |  |  |  |
| Not Altered |  | 1 | 0.42, 2.20 | >0.9 |  |  |  |
| *PIK3CA* Alteration | 80 | 1.6 | 0.73, 3.52 | 0.2 |  |  |  |
| ERBB2 Alteration | 80 |  |  |  |  |  |  |
| Altered |  | — | — |  | — | — |  |
| Not Altered |  | 2.7 | 1.21, 6.06 | **0.015** | 3 | 1.22, 7.34 | **0.016** |
| p53 Pathway | 80 |  |  |  |  |  |  |
| Altered |  | — | — |  |  |  |  |
| Not Altered |  | 0.8 | 0.28, 2.28 | 0.7 |  |  |  |
| PI3K Pathway | 80 |  |  |  |  |  |  |
| Altered |  | — | — |  |  |  |  |
| Not Altered |  | 1.1 | 0.53, 2.30 | 0.8 |  |  |  |
| Cell Cycle Pathway | 80 |  |  |  |  |  |  |
| Altered |  | — | — |  |  |  |  |
| Not Altered |  | 0.7 | 0.33, 1.34 | 0.3 |  |  |  |
| Abbreviations: CI = Confidence Interval, HR = Hazard Ratio, aHR = adjusted Hazard Ratio | | | | |  |  |  |

**Supplemental Table 9.** Univariable and multivariable Cox Proportional Hazard Models for Overall Survival after diagnosis for Early Stage (TNM I-III) HER2+ patients.

|  |  | **Univariable** | | | **Multivariable** | | |
| --- | --- | --- | --- | --- | --- | --- | --- |
| **Characteristic** | **N** | **HR** | **95% CI** | **p-value** | **aHR** | **95% CI** | **p-value** |
| Age at Diagnosis | 150 | 1.1 | 1.01, 1.10 | **0.008** | 1.02 | 0.98, 1.07 | 0.3 |
| Race | 150 |  |  |  |  |  |  |
| White |  | — | — |  | — | — |  |
| Black |  | 1.4 | 0.68, 3.02 | 0.3 | 0.92 | 0.39, 2.17 | 0.9 |
| Asian |  | 0.2 | 0.03, 1.76 | 0.2 | 0.36 | 0.05, 2.80 | 0.3 |
| Other/Unknown |  | 0.6 | 0.18, 1.82 | 0.3 | 0.32 | 0.08, 1.22 | 0.1 |
| Ethnicity | 150 |  |  |  |  |  |  |
| Non-Hispanic |  | — | — |  | — | — |  |
| Hispanic |  | 0.4 | 0.06, 3.09 | 0.4 | 0.5 | 0.06, 3.99 | 0.5 |
| Unknown |  | 0 | 0.00, Inf | >0.9 | 0 | 0.00, Inf | >0.9 |
| Tumor Laterality | 150 |  |  |  |  |  |  |
| Left |  | — | — |  | — | — |  |
| Other/Unknown |  | 1.7 | 0.88, 3.35 | 0.12 | 1.74 | 0.80, 3.81 | 0.2 |
| Right |  | 0.8 | 0.43, 1.33 | 0.3 | 0.6 | 0.31, 1.15 | 0.12 |
| Histology | 150 |  |  |  |  |  |  |
| Invasive ductal carcinoma |  | — | — |  | — | — |  |
| Invasive lobular carcinoma |  | 4.7 | 1.10, 20.1 | **0.037** | 1.56 | 0.34, 7.14 | 0.6 |
| Other histology |  | 4.7 | 1.11, 19.4 | **0.035** | 5.37 | 1.06, 27.2 | **0.043** |
| ER Status | 150 |  |  |  |  |  |  |
| Positive |  | — | — |  |  |  |  |
| Negative |  | 2.7 | 1.59, 4.51 | **<0.001** |  |  |  |
| Borderline |  |  |  |  |  |  |  |
| Unknown/Not Tested |  |  |  |  |  |  |  |
| PR Status | 150 |  |  |  |  |  |  |
| Positive |  | — | — |  |  |  |  |
| Negative |  | 2.4 | 1.46, 4.01 | **<0.001** |  |  |  |
| Unknown/Not Tested |  | 0 | 0.00, Inf | >0.9 |  |  |  |
| Breast Cancer Subtype | 150 |  |  |  |  |  |  |
| HER2+, HR- |  | — | — |  | — | — |  |
| HER2+, HR+ |  | 0.4 | 0.22, 0.62 | **<0.001** | 0.69 | 0.32, 1.53 | 0.4 |
| Received Chemotherapy | 150 |  |  |  |  |  |  |
| Yes |  | — | — |  |  |  |  |
| No |  | 2.4 | 0.32, 17.6 | 0.4 |  |  |  |
| Received Hormone Therapy | 150 |  |  |  |  |  |  |
| Yes |  | — | — |  | — | — |  |
| No |  | 3.3 | 1.89, 5.69 | **<0.001** | 3.17 | 1.36, 7.39 | **0.008** |
| Received Immunotherapy | 150 |  |  |  |  |  |  |
| Yes |  | — | — |  | — | — |  |
| No |  | 0.4 | 0.21, 0.76 | **0.005** | 0.56 | 0.28, 1.13 | 0.11 |
| Received HER2 Therapy | 150 |  |  |  |  |  |  |
| Yes |  | — | — |  | — | — |  |
| No |  | 0.6 | 0.26, 1.22 | 0.15 | 0.41 | 0.16, 1.06 | 0.065 |
| Received CDK4/6 Inhibitors | 150 |  |  |  |  |  |  |
| Yes |  | — | — |  |  |  |  |
| No |  | 1.4 | 0.75, 2.55 | 0.3 |  |  |  |
| Received PI3K Inhibitors | 150 |  |  |  |  |  |  |
| Yes |  | — | — |  |  |  |  |
| No |  | 1.4 | 0.64, 2.87 | 0.4 |  |  |  |
| Received Other Therapy | 150 |  |  |  |  |  |  |
| Yes |  | — | — |  | — | — |  |
| No |  | 2.2 | 0.68, 6.94 | 0.2 | 1.43 | 0.65, 3.14 | 0.4 |
| TP53 Alteration | 150 |  |  |  |  |  |  |
| Altered |  | — | — |  |  |  |  |
| Not Altered |  | 0.8 | 0.46, 1.31 | 0.3 |  |  |  |
| *PIK3CA* Alteration | 150 | 1 | 0.61, 1.74 | 0.9 |  |  |  |
| ERBB2 Alteration | 150 |  |  |  |  |  |  |
| Altered |  | — | — |  | — | — |  |
| Not Altered |  | 1.3 | 0.80, 2.22 | 0.3 | 2.24 | 1.20, 4.16 | **0.011** |
| p53 Pathway | 150 |  |  |  |  |  |  |
| Altered |  | — | — |  |  |  |  |
| Not Altered |  | 0.8 | 0.46, 1.45 | 0.5 |  |  |  |
| PI3K Pathway | 150 |  |  |  |  |  |  |
| Altered |  | — | — |  |  |  |  |
| Not Altered |  | 1.4 | 0.81, 2.23 | 0.2 |  |  |  |
| Cell Cycle Pathway | 150 |  |  |  |  |  |  |
| Altered |  | — | — |  |  |  |  |
| Not Altered |  | 0.9 | 0.52, 1.48 | 0.6 |  |  |  |
| Abbreviations: CI = Confidence Interval, HR = Hazard Ratio, aHR = adjusted Hazard Ratio | | | | |  |  |  |

**Supplemental Table 10.** Univariable and multivariable Cox Proportional Hazard Models for Overall Survival after diagnosis for Stage IV HER2+ patients.

|  |  | **Univariable** | | | **Multivariable** | | |
| --- | --- | --- | --- | --- | --- | --- | --- |
| **Characteristic** | **N** | **HR** | **95% CI** | **p-value** | **aHR** | **95% CI** | **p-value** |
| Age at Diagnosis | 62 | 1 | 0.94, 1.08 | 0.8 |  |  |  |
| Race | 62 |  |  |  |  |  |  |
| White |  | — | — |  |  |  |  |
| Black |  | 1.1 | 0.15, 8.68 | >0.9 |  |  |  |
| Asian |  | 4.6 | 1.01, 20.6 | **0.048** |  |  |  |
| Other/Unknown |  | 4.5 | 0.56, 36.1 | 0.2 |  |  |  |
| Ethnicity | 62 |  |  |  |  |  |  |
| Non-Hispanic |  | — | — |  |  |  |  |
| Hispanic |  | 3.5 | 0.44, 28.5 | 0.2 |  |  |  |
| Unknown |  | 4.1 | 0.52, 32.4 | 0.2 |  |  |  |
| Tumor Laterality | 62 |  |  |  |  |  |  |
| Left |  | — | — |  |  |  |  |
| Other/Unknown |  | 0.9 | 0.29, 2.96 | >0.9 |  |  |  |
| Right |  | 0.8 | 0.30, 2.04 | 0.6 |  |  |  |
| Histology | 62 |  |  |  |  |  |  |
| Invasive ductal carcinoma |  | — | — |  |  |  |  |
| Invasive lobular carcinoma |  | 0.9 | 0.12, 6.66 | 0.9 |  |  |  |
| Other histology |  |  |  |  |  |  |  |
| ER Status | 62 |  |  |  |  |  |  |
| Positive |  | — | — |  |  |  |  |
| Negative |  | 1.4 | 0.60, 3.21 | 0.4 |  |  |  |
| Borderline |  |  |  |  |  |  |  |
| Unknown/Not Tested |  |  |  |  |  |  |  |
| PR Status | 62 |  |  |  |  |  |  |
| Positive |  | — | — |  |  |  |  |
| Negative |  | 1.6 | 0.69, 3.77 | 0.3 |  |  |  |
| Unknown/Not Tested |  |  |  |  |  |  |  |
| Breast Cancer Subtype | 62 |  |  |  |  |  |  |
| HER2+, HR- |  | — | — |  |  |  |  |
| HER2+, HR+ |  | 0.6 | 0.24, 1.30 | 0.2 |  |  |  |
| Received Chemotherapy | 62 |  |  |  |  |  |  |
| Yes |  | — | — |  |  |  |  |
| No |  | 2.1 | 0.28, 16.3 | 0.5 |  |  |  |
| Received Hormone Therapy | 62 |  |  |  |  |  |  |
| Yes |  | — | — |  |  |  |  |
| No |  | 2 | 0.82, 4.67 | 0.13 |  |  |  |
| Received Immunotherapy | 62 |  |  |  |  |  |  |
| Yes |  | — | — |  |  |  |  |
| No |  | 0.4 | 0.05, 2.85 | 0.3 |  |  |  |
| Received HER2 Therapy | 62 |  |  |  |  |  |  |
| Yes |  | — | — |  |  |  |  |
| No |  | 0.7 | 0.10, 5.71 | 0.8 |  |  |  |
| Received CDK4/6 Inhibitors | 62 |  |  |  |  |  |  |
| Yes |  | — | — |  |  |  |  |
| No |  | 0.9 | 0.27, 3.21 | >0.9 |  |  |  |
| Received PI3K Inhibitors | 62 |  |  |  |  |  |  |
| Yes |  | — | — |  |  |  |  |
| No |  | 0.9 | 0.26, 3.14 | 0.9 |  |  |  |
| Received Other Therapy | 62 |  |  |  |  |  |  |
| Yes |  | — | — |  |  |  |  |
| No |  | ### | 0.00, Inf | >0.9 |  |  |  |
| TP53 Alteration | 62 |  |  |  |  |  |  |
| Altered |  | — | — |  |  |  |  |
| Not Altered |  | 0.4 | 0.16, 1.17 | 0.1 |  |  |  |
| *PIK3CA* Alteration | 62 | 1.3 | 0.52, 3.16 | 0.6 |  |  |  |
| ERBB2 Alteration | 62 |  |  |  |  |  |  |
| Altered |  | — | — |  |  |  |  |
| Not Altered |  | 1.2 | 0.43, 3.24 | 0.7 |  |  |  |
| p53 Pathway | 62 |  |  |  |  |  |  |
| Altered |  | — | — |  |  |  |  |
| Not Altered |  | 0.6 | 0.19, 1.71 | 0.3 |  |  |  |
| PI3K Pathway | 62 |  |  |  |  |  |  |
| Altered |  | — | — |  |  |  |  |
| Not Altered |  | 1.1 | 0.44, 2.55 | 0.9 |  |  |  |
| Cell Cycle Pathway | 62 |  |  |  |  |  |  |
| Altered |  | — | — |  |  |  |  |
| Not Altered |  | 0.7 | 0.25, 1.87 | 0.5 |  |  |  |
| Abbreviations: CI = Confidence Interval, HR = Hazard Ratio, aHR = adjusted Hazard Ratio | | | | | | | |

**Supplemental Table 11.** Univariable and multivariable Cox Proportional Hazard Models for Overall Survival after treatment for HER2+/ER+ patients.

|  |  | **Univariable** | | | **Multivariable** | | |
| --- | --- | --- | --- | --- | --- | --- | --- |
| **Characteristic** | **N** | **HR** | **95% CI** | **p-value** | **aHR** | **95% CI** | **p-value** |
| Age at Diagnosis | 131 | 1.1 | 1.00, 1.10 | **0.032** | 1.04 | 0.99, 1.09 | 0.1 |
| Race | 131 |  |  |  |  |  |  |
| White |  | — | — |  |  |  |  |
| Black |  | 0.9 | 0.22, 3.79 | >0.9 |  |  |  |
| Asian |  | 0.6 | 0.15, 2.57 | 0.5 |  |  |  |
| Other/Unknown |  | 0.9 | 0.12, 6.55 | >0.9 |  |  |  |
| Ethnicity | 131 |  |  |  |  |  |  |
| Non-Hispanic |  | — | — |  |  |  |  |
| Hispanic |  | 1.5 | 0.36, 6.28 | 0.6 |  |  |  |
| Unknown |  | 0 | 0.00, Inf | >0.9 |  |  |  |
| Tumor Laterality | 131 |  |  |  |  |  |  |
| Left |  | — | — |  | — | — |  |
| Other/Unknown |  | 1.3 | 0.55, 2.97 | 0.6 | 1.22 | 0.48, 3.09 | 0.7 |
| Right |  | 0.6 | 0.32, 1.17 | 0.14 | 0.54 | 0.27, 1.08 | 0.083 |
| Histology | 131 |  |  |  |  |  |  |
| Invasive ductal carcinoma |  | — | — |  | — | — |  |
| Invasive lobular carcinoma |  | 2.9 | 0.69, 12.1 | 0.15 | 2.51 | 0.56, 11.3 | 0.2 |
| Other histology |  | 4.4 | 0.59, 32.9 | 0.15 | 15.1 | 1.64, 140 | **0.017** |
| PR Status | 131 |  |  |  |  |  |  |
| Positive |  | — | — |  |  |  |  |
| Negative |  | 1.5 | 0.65, 3.26 | 0.4 |  |  |  |
| Unknown/Not Tested |  | 0 | 0.00, Inf | >0.9 |  |  |  |
| Stage at Diagnosis | 131 |  |  |  |  |  |  |
| Early Stage (I-III) |  | — | — |  |  |  |  |
| Stage IV |  | 1.5 | 0.73, 2.90 | 0.3 |  |  |  |
| Unknown/Other Stage |  |  |  |  |  |  |  |
| Received Chemotherapy | 131 |  |  |  |  |  |  |
| Yes |  | — | — |  |  |  |  |
| No |  | 0 | 0.00, Inf | >0.9 |  |  |  |
| Received Hormone Therapy | 131 |  |  |  |  |  |  |
| Yes |  | — | — |  |  |  |  |
| No |  | 2.6 | 0.60, 11.3 | 0.2 |  |  |  |
| Received Immunotherapy | 131 |  |  |  |  |  |  |
| Yes |  | — | — |  |  |  |  |
| No |  | 0.6 | 0.23, 1.50 | 0.3 |  |  |  |
| Received HER2 Therapy | 131 |  |  |  |  |  |  |
| Yes |  | — | — |  | — | — |  |
| No |  | 0.5 | 0.22, 1.19 | 0.12 | 0.25 | 0.09, 0.69 | **0.007** |
| Received CDK4/6 Inhibitors | 131 |  |  |  |  |  |  |
| Yes |  | — | — |  |  |  |  |
| No |  | 1.1 | 0.57, 2.00 | 0.8 |  |  |  |
| Received PI3K Inhibitors | 131 |  |  |  |  |  |  |
| Yes |  | — | — |  |  |  |  |
| No |  | 1 | 0.48, 1.98 | >0.9 |  |  |  |
| Received Other Therapy | 131 |  |  |  |  |  |  |
| Yes |  | — | — |  |  |  |  |
| No |  | 2.1 | 0.51, 8.89 | 0.3 |  |  |  |
| TP53 Alteration | 131 |  |  |  |  |  |  |
| Altered |  | — | — |  |  |  |  |
| Not Altered |  | 0.8 | 0.42, 1.35 | 0.3 |  |  |  |
| *PIK3CA* Alteration | 131 | 1.2 | 0.67, 2.10 | 0.6 |  |  |  |
| ERBB2 Alteration | 131 |  |  |  |  |  |  |
| Altered |  | — | — |  | — | — |  |
| Not Altered |  | 1.3 | 0.74, 2.37 | 0.3 | 1.96 | 0.96, 3.97 | 0.063 |
| p53 Pathway | 131 |  |  |  |  |  |  |
| Altered |  | — | — |  |  |  |  |
| Not Altered |  | 0.9 | 0.49, 1.65 | 0.7 |  |  |  |
| PI3K Pathway | 131 |  |  |  |  |  |  |
| Altered |  | — | — |  |  |  |  |
| Not Altered |  | 1.1 | 0.63, 1.96 | 0.7 |  |  |  |
| Cell Cycle Pathway | 131 |  |  |  |  |  |  |
| Altered |  | — | — |  |  |  |  |
| Not Altered |  | 1 | 0.54, 1.90 | >0.9 |  |  |  |

Abbreviations: CI = Confidence Interval, HR = Hazard Ratio, aHR = adjusted Hazard Ratio

**Supplemental Table 12.** Univariable and multivariable Cox Proportional Hazard Models for Overall Survival after treatment for HER2+/ER- patients.

|  |  | **Univariable** | | | **Multivariable** | | |
| --- | --- | --- | --- | --- | --- | --- | --- |
| **Characteristic** | **N** | **HR** | **95% CI** | **p-value** | **aHR** | **95% CI** | **p-value** |
| Age at Diagnosis | 78 | 1 | 0.96, 1.07 | 0.7 |  |  |  |
| Race | 78 |  |  |  |  |  |  |
| White |  | — | — |  |  |  |  |
| Black |  | 1.1 | 0.49, 2.69 | 0.8 |  |  |  |
| Asian |  | 1 | 0.14, 7.73 | >0.9 |  |  |  |
| Other/Unknown |  | 0.7 | 0.19, 2.18 | 0.5 |  |  |  |
| Ethnicity | 78 |  |  |  |  |  |  |
| Non-Hispanic |  | — | — |  |  |  |  |
| Hispanic |  | 0 | 0.00, Inf | >0.9 |  |  |  |
| Unknown |  | 2.1 | 0.28, 15.7 | 0.5 |  |  |  |
| Tumor Laterality | 78 |  |  |  |  |  |  |
| Left |  | — | — |  |  |  |  |
| Other/Unknown |  | 0.9 | 0.37, 2.43 | >0.9 |  |  |  |
| Right |  | 1.1 | 0.52, 2.37 | 0.8 |  |  |  |
| Histology | 78 |  |  |  |  |  |  |
| Invasive ductal carcinoma |  | — | — |  |  |  |  |
| Invasive lobular carcinoma |  | 1.9 | 0.26, 14.5 | 0.5 |  |  |  |
| Other histology |  | 6.1 | 0.78, 47.2 | 0.085 |  |  |  |
| PR Status | 78 |  |  |  |  |  |  |
| Positive |  | — | — |  |  |  |  |
| Negative |  | ### | 0.00, Inf | >0.9 |  |  |  |
| Unknown/Not Tested |  |  |  |  |  |  |  |
| Stage at Diagnosis | 78 |  |  |  |  |  |  |
| Early Stage (I-III) |  | — | — |  |  |  |  |
| Stage IV |  | 0.6 | 0.27, 1.26 | 0.2 |  |  |  |
| Unknown/Other Stage |  |  |  |  |  |  |  |
| Breast Cancer Subtype | 78 |  |  |  |  |  |  |
| HER2+, HR- |  | — | — |  |  |  |  |
| HER2+, HR+ |  | 0 | 0.00, Inf | >0.9 |  |  |  |
| Received Chemotherapy | 78 |  |  |  |  |  |  |
| Yes |  | — | — |  |  |  |  |
| No |  | 0 | 0.00, Inf | >0.9 |  |  |  |
| Received Hormone Therapy | 78 |  |  |  |  |  |  |
| Yes |  | — | — |  | — | — |  |
| No |  | 2.6 | 1.13, 5.79 | **0.025** | 2.06 | 0.88, 4.82 | 0.1 |
| Received Immunotherapy | 78 |  |  |  |  |  |  |
| Yes |  | — | — |  | — | — |  |
| No |  | 0.4 | 0.18, 0.91 | **0.029** | 0.35 | 0.15, 0.79 | **0.012** |
| Received CDK4/6 Inhibitors | 78 |  |  |  |  |  |  |
| Yes |  | — | — |  | — | — |  |
| No |  | 3.6 | 0.48, 26.7 | 0.2 | 3.42 | 0.41, 28.7 | 0.3 |
| Received PI3K Inhibitors | 78 |  |  |  |  |  |  |
| Yes |  | — | — |  |  |  |  |
| No |  | ### | 0.00, Inf | >0.9 |  |  |  |
| Received Other Therapy | 78 |  |  |  |  |  |  |
| Yes |  | — | — |  |  |  |  |
| No |  | 2.1 | 0.28, 16.1 | 0.5 |  |  |  |
| TP53 Alteration | 78 |  |  |  |  |  |  |
| Altered |  | — | — |  |  |  |  |
| Not Altered |  | 0.7 | 0.30, 1.74 | 0.5 |  |  |  |
| *PIK3CA* Alteration | 78 | 1.6 | 0.71, 3.76 | 0.3 |  |  |  |
| ERBB2 Alteration | 78 |  |  |  |  |  |  |
| Altered |  | — | — |  |  |  |  |
| Not Altered |  | 1.7 | 0.75, 4.05 | 0.2 |  |  |  |
| p53 Pathway | 78 |  |  |  |  |  |  |
| Altered |  | — | — |  |  |  |  |
| Not Altered |  | 0.8 | 0.27, 2.23 | 0.6 |  |  |  |
| PI3K Pathway | 78 |  |  |  |  |  |  |
| Altered |  | — | — |  |  |  |  |
| Not Altered |  | 1 | 0.47, 2.20 | >0.9 |  |  |  |
| Cell Cycle Pathway | 78 |  |  |  |  |  |  |
| Altered |  | — | — |  |  |  |  |
| Not Altered |  | 0.7 | 0.36, 1.53 | 0.4 |  |  |  |

Abbreviations: CI = Confidence Interval, HR = Hazard Ratio, aHR = adjusted Hazard Ratio

**Supplemental Table 13.** Univariable and multivariable Cox Proportional Hazard Models for Overall Survival after treatment for Early Stage HER2+ patients.

|  |  | **Univariable** | | | **Multivariable** | | |
| --- | --- | --- | --- | --- | --- | --- | --- |
| **Characteristic** | **N** | **HR** | **95% CI** | **p-value** | **aHR** | **95% CI** | **p-value** |
| Age at Diagnosis | 148 | 1.1 | 1.01, 1.10 | **0.01** | 1.04 | 1.00, 1.08 | 0.068 |
| Race | 148 |  |  |  |  |  |  |
| White |  | — | — |  |  |  |  |
| Black |  | 1.4 | 0.65, 2.89 | 0.4 |  |  |  |
| Asian |  | 0.2 | 0.03, 1.76 | 0.2 |  |  |  |
| Other/Unknown |  | 0.6 | 0.20, 2.06 | 0.5 |  |  |  |
| Ethnicity | 148 |  |  |  |  |  |  |
| Non-Hispanic |  | — | — |  |  |  |  |
| Hispanic |  | 0.4 | 0.06, 3.22 | 0.4 |  |  |  |
| Unknown |  | 0 | 0.00, Inf | >0.9 |  |  |  |
| Tumor Laterality | 148 |  |  |  |  |  |  |
| Left |  | — | — |  | — | — |  |
| Other/Unknown |  | 1.7 | 0.85, 3.40 | 0.13 | 1.93 | 0.93, 3.98 | 0.077 |
| Right |  | 0.7 | 0.41, 1.30 | 0.3 | 0.76 | 0.42, 1.39 | 0.4 |
| Histology | 148 |  |  |  |  |  |  |
| Invasive ductal carcinoma |  | — | — |  | — | — |  |
| Invasive lobular carcinoma |  | 4.3 | 1.01, 18.3 | **0.048** | 1.66 | 0.37, 7.58 | 0.5 |
| Other histology |  | 4.9 | 1.17, 20.5 | **0.03** | 9.63 | 2.06, 45.0 | **0.004** |
| ER Status | 148 |  |  |  |  |  |  |
| Positive |  | — | — |  |  |  |  |
| Negative |  | 2.5 | 1.49, 4.24 | **<0.001** |  |  |  |
| Borderline |  |  |  |  |  |  |  |
| Unknown/Not Tested |  |  |  |  |  |  |  |
| PR Status | 148 |  |  |  |  |  |  |
| Positive |  | — | — |  |  |  |  |
| Negative |  | 2.3 | 1.39, 3.83 | **0.001** |  |  |  |
| Unknown/Not Tested |  | 0 | 0.00, Inf | >0.9 |  |  |  |
| Breast Cancer Subtype | 148 |  |  |  |  |  |  |
| HER2+, HR- |  | — | — |  | — | — |  |
| HER2+, HR+ |  | 0.4 | 0.23, 0.67 | **<0.001** | 0.93 | 0.42, 2.07 | 0.9 |
| Received Chemotherapy | 148 |  |  |  |  |  |  |
| Yes |  | — | — |  |  |  |  |
| No |  | 0 | 0.00, Inf | >0.9 |  |  |  |
| Received Hormone Therapy | 148 |  |  |  |  |  |  |
| Yes |  | — | — |  | — | — |  |
| No |  | 3.3 | 1.86, 5.73 | **<0.001** | 2.83 | 1.19, 6.76 | **0.019** |
| Received Immunotherapy | 148 |  |  |  |  |  |  |
| Yes |  | — | — |  | — | — |  |
| No |  | 0.4 | 0.22, 0.80 | **0.008** | 0.45 | 0.23, 0.90 | **0.023** |
| Received HER2 Therapy | 148 |  |  |  |  |  |  |
| Yes |  | — | — |  |  |  |  |
| No |  | 0.5 | 0.23, 1.15 | 0.11 |  |  |  |
| Received CDK4/6 Inhibitors | 148 |  |  |  |  |  |  |
| Yes |  | — | — |  |  |  |  |
| No |  | 1.4 | 0.74, 2.53 | 0.3 |  |  |  |
| Received PI3K Inhibitors | 148 |  |  |  |  |  |  |
| Yes |  | — | — |  |  |  |  |
| No |  | 1.3 | 0.61, 2.71 | 0.5 |  |  |  |
| Received Other Therapy | 148 |  |  |  |  |  |  |
| Yes |  | — | — |  |  |  |  |
| No |  | 2.1 | 0.66, 6.78 | 0.2 |  |  |  |
| TP53 Alteration | 148 |  |  |  |  |  |  |
| Altered |  | — | — |  |  |  |  |
| Not Altered |  | 0.8 | 0.46, 1.32 | 0.4 |  |  |  |
| *PIK3CA* Alteration | 148 | 1 | 0.60, 1.72 | >0.9 |  |  |  |
| ERBB2 Alteration | 148 |  |  |  |  |  |  |
| Altered |  | — | — |  |  |  |  |
| Not Altered |  | 1.3 | 0.80, 2.25 | 0.3 |  |  |  |
| p53 Pathway | 148 |  |  |  |  |  |  |
| Altered |  | — | — |  |  |  |  |
| Not Altered |  | 0.8 | 0.46, 1.46 | 0.5 |  |  |  |
| PI3K Pathway | 148 |  |  |  |  |  |  |
| Altered |  | — | — |  |  |  |  |
| Not Altered |  | 1.3 | 0.81, 2.24 | 0.3 |  |  |  |
| Cell Cycle Pathway | 148 |  |  |  |  |  |  |
| Altered |  | — | — |  |  |  |  |
| Not Altered |  | 0.9 | 0.51, 1.48 | 0.6 |  |  |  |

Abbreviations: CI = Confidence Interval, HR = Hazard Ratio, aHR = adjusted Hazard Ratio

**Supplemental Table 14.** Univariable and multivariable Cox Proportional Hazard Models for Overall Survival after treatment for Stage IV HER2+ patients.

|  |  | **Univariable** | | | **Multivariable** | | |
| --- | --- | --- | --- | --- | --- | --- | --- |
| **Characteristic** | **N** | **HR** | **95% CI** | **p-value** | **aHR** | **95% CI** | **p-value** |
| Age at Diagnosis | 61 | 1.01 | 0.94, 1.08 | 0.8 |  |  |  |
| Race | 61 |  |  |  |  |  |  |
| White |  | — | — |  |  |  |  |
| Black |  | 1.32 | 0.17, 10.2 | 0.8 |  |  |  |
| Asian |  | 4.69 | 1.03, 21.3 | **0.046** |  |  |  |
| Other/Unknown |  | 5.13 | 0.63, 41.9 | 0.13 |  |  |  |
| Ethnicity | 61 |  |  |  |  |  |  |
| Non-Hispanic |  | — | — |  |  |  |  |
| Hispanic |  | 3.63 | 0.45, 29.3 | 0.2 |  |  |  |
| Unknown |  | 4.47 | 0.56, 35.5 | 0.2 |  |  |  |
| Tumor Laterality | 61 |  |  |  |  |  |  |
| Left |  | — | — |  |  |  |  |
| Other/Unknown |  | 0.44 | 0.10, 2.01 | 0.3 |  |  |  |
| Right |  | 0.79 | 0.31, 2.06 | 0.6 |  |  |  |
| Histology | 61 |  |  |  |  |  |  |
| Invasive ductal carcinoma |  | — | — |  |  |  |  |
| Invasive lobular carcinoma |  | 1.04 | 0.14, 7.92 | >0.9 |  |  |  |
| Other histology |  |  |  |  |  |  |  |
| ER Status | 61 |  |  |  |  |  |  |
| Positive |  | — | — |  |  |  |  |
| Negative |  | 1.08 | 0.45, 2.63 | 0.9 |  |  |  |
| Borderline |  |  |  |  |  |  |  |
| Unknown/Not Tested |  |  |  |  |  |  |  |
| PR Status | 61 |  |  |  |  |  |  |
| Positive |  | — | — |  |  |  |  |
| Negative |  | 1.6 | 0.65, 3.93 | 0.3 |  |  |  |
| Unknown/Not Tested |  |  |  |  |  |  |  |
| Breast Cancer Subtype | 61 |  |  |  |  |  |  |
| HER2+, HR- |  | — | — |  |  |  |  |
| HER2+, HR+ |  | 0.62 | 0.25, 1.50 | 0.3 |  |  |  |
| Received Chemotherapy | 61 |  |  |  |  |  |  |
| Yes |  | — | — |  |  |  |  |
| No |  | 0 | 0.00, Inf | >0.9 |  |  |  |
| Received Hormone Therapy | 61 |  |  |  |  |  |  |
| Yes |  | — | — |  |  |  |  |
| No |  | 1.92 | 0.77, 4.79 | 0.2 |  |  |  |
| Received Immunotherapy | 61 |  |  |  |  |  |  |
| Yes |  | — | — |  |  |  |  |
| No |  | 0.33 | 0.04, 2.60 | 0.3 |  |  |  |
| Received HER2 Therapy | 61 |  |  |  |  |  |  |
| Yes |  | — | — |  |  |  |  |
| No |  | 0 | 0.00, Inf | >0.9 |  |  |  |
| Received CDK4/6 Inhibitors | 61 |  |  |  |  |  |  |
| Yes |  | — | — |  |  |  |  |
| No |  | 1.49 | 0.34, 6.65 | 0.6 |  |  |  |
| Received PI3K Inhibitors | 61 |  |  |  |  |  |  |
| Yes |  | — | — |  |  |  |  |
| No |  | 1.48 | 0.33, 6.57 | 0.6 |  |  |  |
| Received Other Therapy | 61 |  |  |  |  |  |  |
| Yes |  | — | — |  |  |  |  |
| No |  | 9,224,899 | 0.00, Inf | >0.9 |  |  |  |
| TP53 Alteration | 61 |  |  |  |  |  |  |
| Altered |  | — | — |  |  |  |  |
| Not Altered |  | 0.36 | 0.12, 1.07 | 0.067 |  |  |  |
| *PIK3CA* Alteration | 61 | 1.53 | 0.60, 3.85 | 0.4 |  |  |  |
| ERBB2 Alteration | 61 |  |  |  |  |  |  |
| Altered |  | — | — |  |  |  |  |
| Not Altered |  | 0.95 | 0.31, 2.90 | >0.9 |  |  |  |
| p53 Pathway | 61 |  |  |  |  |  |  |
| Altered |  | — | — |  |  |  |  |
| Not Altered |  | 0.67 | 0.22, 2.01 | 0.5 |  |  |  |
| PI3K Pathway | 61 |  |  |  |  |  |  |
| Altered |  | — | — |  |  |  |  |
| Not Altered |  | 0.87 | 0.35, 2.15 | 0.8 |  |  |  |
| Cell Cycle Pathway | 61 |  |  |  |  |  |  |
| Altered |  | — | — |  |  |  |  |
| Not Altered |  | 0.85 | 0.28, 2.59 | 0.8 |  |  |  |

Abbreviations: CI = Confidence Interval, HR = Hazard Ratio, aHR = adjusted Hazard Ratio

**Supplemental Table 15.** Univariable and multivariable Cox Proportional Hazard Models for Distant Recurrence-Free Survival after diagnosis for Early Stage HER2+ patients.

|  |  | **Univariable** | | | **Multivariable** | | |
| --- | --- | --- | --- | --- | --- | --- | --- |
| **Characteristic** | **N** | **HR** | **95% CI** | **p-value** | **aHR** | **95% CI** | **p-value** |
| Age at Diagnosis | 115 | 1.05 | 1.02, 1.08 | **0.002** | 1.03 | 1.00, 1.06 | 0.053 |
| Race | 115 |  |  |  |  |  |  |
| White |  | — | — |  |  |  |  |
| Black |  | 1.23 | 0.65, 2.31 | 0.5 |  |  |  |
| Asian |  | 0.53 | 0.21, 1.32 | 0.2 |  |  |  |
| Other/Unknown |  | 1.63 | 0.91, 2.89 | 0.1 |  |  |  |
| Ethnicity | 115 |  |  |  |  |  |  |
| Non-Hispanic |  | — | — |  |  |  |  |
| Hispanic |  | 0.93 | 0.29, 2.93 | 0.9 |  |  |  |
| Unknown |  | 1.32 | 0.42, 4.17 | 0.6 |  |  |  |
| Tumor Laterality | 115 |  |  |  |  |  |  |
| Left |  | — | — |  |  |  |  |
| Other/Unknown |  | 1.36 | 0.81, 2.28 | 0.3 |  |  |  |
| Right |  | 0.95 | 0.63, 1.43 | 0.8 |  |  |  |
| Histology | 115 |  |  |  |  |  |  |
| Invasive ductal carcinoma |  | — | — |  |  |  |  |
| Invasive lobular carcinoma |  | 2.81 | 0.68, 11.6 | 0.2 |  |  |  |
| Other histology |  | 1.38 | 0.34, 5.65 | 0.7 |  |  |  |
| ER Status | 115 |  |  |  |  |  |  |
| Positive |  | — | — |  |  |  |  |
| Negative |  | 2.22 | 1.48, 3.34 | **<0.001** |  |  |  |
| Borderline |  |  |  |  |  |  |  |
| Unknown/Not Tested |  |  |  |  |  |  |  |
| PR Status | 115 |  |  |  |  |  |  |
| Positive |  | — | — |  |  |  |  |
| Negative |  | 1.89 | 1.28, 2.78 | **0.001** |  |  |  |
| Unknown/Not Tested |  | 1.62 | 0.22, 11.8 | 0.6 |  |  |  |
| Breast Cancer Subtype | 115 |  |  |  |  |  |  |
| HER2+, HR- |  | — | — |  |  |  |  |
| HER2+, HR+ |  | 0.45 | 0.30, 0.68 | **<0.001** |  |  |  |
| Received Hormone Therapy | 115 |  |  |  |  |  |  |
| Yes |  | — | — |  | — | — |  |
| No |  | 2.86 | 1.84, 4.43 | **<0.001** | 2.1 | 1.31, 3.37 | **0.002** |
| Received Immunotherapy | 115 |  |  |  |  |  |  |
| Yes |  | — | — |  |  |  |  |
| No |  | 0.63 | 0.38, 1.07 | 0.086 |  |  |  |
| Received HER2 Therapy | 115 |  |  |  |  |  |  |
| Yes |  | — | — |  | — | — |  |
| No |  | 0.35 | 0.18, 0.68 | **0.002** | 0.5 | 0.25, 1.03 | 0.059 |
| Received CDK4/6 Inhibitors | 115 |  |  |  |  |  |  |
| Yes |  | — | — |  | — | — |  |
| No |  | 2.05 | 1.28, 3.28 | **0.003** | 1.43 | 0.85, 2.40 | 0.2 |
| Received PI3K Inhibitors | 115 |  |  |  |  |  |  |
| Yes |  | — | — |  |  |  |  |
| No |  | 1.29 | 0.72, 2.29 | 0.4 |  |  |  |
| Received Other Therapy | 115 |  |  |  |  |  |  |
| Yes |  | — | — |  |  |  |  |
| No |  | 1.43 | 0.86, 2.38 | 0.2 |  |  |  |
| TP53 Alteration | 115 |  |  |  |  |  |  |
| Altered |  | — | — |  |  |  |  |
| Not Altered |  | 0.62 | 0.42, 0.92 | **0.017** |  |  |  |
| *PIK3CA* Alteration | 115 | 0.67 | 0.44, 1.00 | 0.052 |  |  |  |
| ERBB2 Alteration | 115 |  |  |  |  |  |  |
| Altered |  | — | — |  |  |  |  |
| Not Altered |  | 0.69 | 0.46, 1.04 | 0.074 |  |  |  |
| p53 Pathway | 115 |  |  |  |  |  |  |
| Altered |  | — | — |  |  |  |  |
| Not Altered |  | 0.56 | 0.36, 0.87 | **0.01** |  |  |  |
| PI3K Pathway | 115 |  |  |  |  |  |  |
| Altered |  | — | — |  |  |  |  |
| Not Altered |  | 1.4 | 0.96, 2.02 | 0.079 |  |  |  |
| Cell Cycle Pathway | 115 |  |  |  |  |  |  |
| Altered |  | — | — |  |  |  |  |
| Not Altered |  | 1.12 | 0.76, 1.66 | 0.6 |  |  |  |

Abbreviations: CI = Confidence Interval, HR = Hazard Ratio, aHR = adjusted Hazard Ratio

**Supplemental Table 16.** Univariable and multivariable Cox Proportional Hazard Models for Progression Free Survival after diagnosis for Stage IV HER2+ patients.

|  |  | **Univariable** | | | **Multivariable** | | |
| --- | --- | --- | --- | --- | --- | --- | --- |
| **Characteristic** | **N** | **HR** | **95% CI** | **p-value** | **aHR** | **95% CI** | **p-value** |
| Age at Diagnosis | 62 | 1.02 | 0.98, 1.06 | 0.2 |  |  |  |
| Race | 62 |  |  |  |  |  |  |
| White |  | — | — |  |  |  |  |
| Black |  | 1.68 | 0.52, 5.45 | 0.4 |  |  |  |
| Asian |  | 1.13 | 0.35, 3.65 | 0.8 |  |  |  |
| Other/Unknown |  | 1.6 | 0.38, 6.68 | 0.5 |  |  |  |
| Ethnicity | 62 |  |  |  |  |  |  |
| Non-Hispanic |  | — | — |  |  |  |  |
| Hispanic |  | 1.13 | 0.27, 4.66 | 0.9 |  |  |  |
| Unknown |  | 3.3 | 0.44, 24.9 | 0.2 |  |  |  |
| Tumor Laterality | 62 |  |  |  |  |  |  |
| Left |  | — | — |  |  |  |  |
| Other/Unknown |  | 0.91 | 0.46, 1.81 | 0.8 |  |  |  |
| Right |  | 0.73 | 0.40, 1.34 | 0.3 |  |  |  |
| Histology | 62 |  |  |  |  |  |  |
| Invasive ductal carcinoma |  | — | — |  |  |  |  |
| Invasive lobular carcinoma |  | 1.27 | 0.39, 4.13 | 0.7 |  |  |  |
| Other histology |  |  |  |  |  |  |  |
| ER Status | 62 |  |  |  |  |  |  |
| Positive |  | — | — |  |  |  |  |
| Negative |  | 0.78 | 0.45, 1.34 | 0.4 |  |  |  |
| Borderline |  |  |  |  |  |  |  |
| Unknown/Not Tested |  |  |  |  |  |  |  |
| PR Status | 62 |  |  |  |  |  |  |
| Positive |  | — | — |  |  |  |  |
| Negative |  | 0.93 | 0.55, 1.59 | 0.8 |  |  |  |
| Unknown/Not Tested |  |  |  |  |  |  |  |
| Breast Cancer Subtype | 62 |  |  |  |  |  |  |
| HER2+, HR- |  | — | — |  |  |  |  |
| HER2+, HR+ |  | 1.25 | 0.71, 2.21 | 0.4 |  |  |  |
| Received Chemotherapy | 62 |  |  |  |  |  |  |
| Yes |  | — | — |  |  |  |  |
| No |  | 1.31 | 0.32, 5.41 | 0.7 |  |  |  |
| Received Hormone Therapy | 62 |  |  |  |  |  |  |
| Yes |  | — | — |  |  |  |  |
| No |  | 0.84 | 0.48, 1.45 | 0.5 |  |  |  |
| Received Immunotherapy | 62 |  |  |  |  |  |  |
| Yes |  | — | — |  |  |  |  |
| No |  | 0.09 | 0.02, 0.44 | **0.003** |  |  |  |
| Received HER2 Therapy | 62 |  |  |  |  |  |  |
| Yes |  | — | — |  |  |  |  |
| No |  | 0.42 | 0.10, 1.77 | 0.2 |  |  |  |
| Received CDK4/6 Inhibitors | 62 |  |  |  |  |  |  |
| Yes |  | — | — |  |  |  |  |
| No |  | 0.77 | 0.26, 2.28 | 0.6 |  |  |  |
| Received PI3K Inhibitors | 62 |  |  |  |  |  |  |
| Yes |  | — | — |  |  |  |  |
| No |  | 1.23 | 0.43, 3.53 | 0.7 |  |  |  |
| Received Other Therapy | 62 |  |  |  |  |  |  |
| Yes |  | — | — |  |  |  |  |
| No |  | 1.48 | 0.20, 10.8 | 0.7 |  |  |  |
| TP53 Alteration | 62 |  |  |  |  |  |  |
| Altered |  | — | — |  |  |  |  |
| Not Altered |  | 1.03 | 0.60, 1.79 | >0.9 |  |  |  |
| *PIK3CA* Alteration | 62 | 1.44 | 0.79, 2.63 | 0.2 |  |  |  |
| ERBB2 Alteration | 62 |  |  |  |  |  |  |
| Altered |  | — | — |  |  |  |  |
| Not Altered |  | 1.23 | 0.61, 2.46 | 0.6 |  |  |  |
| p53 Pathway | 62 |  |  |  |  |  |  |
| Altered |  | — | — |  |  |  |  |
| Not Altered |  | 1.07 | 0.59, 1.95 | 0.8 |  |  |  |
| PI3K Pathway | 62 |  |  |  |  |  |  |
| Altered |  | — | — |  |  |  |  |
| Not Altered |  | 1.12 | 0.65, 1.93 | 0.7 |  |  |  |
| Cell Cycle Pathway | 62 |  |  |  |  |  |  |
| Altered |  | — | — |  |  |  |  |
| Not Altered |  | 0.86 | 0.45, 1.64 | 0.6 |  |  |  |

Abbreviations: CI = Confidence Interval, HR = Hazard Ratio, aHR = adjusted Hazard Ratio

**Supplemental Table 17.** Multivariable Cox Proportional Hazard Models for Overall Survival after diagnosis for HER2+ patients with *PIK3CA* mutation overall and by line of therapy.

|  | **HER2+ *PIK3CA*m** | | | **HER2+ *PIK3CA*m (1st line)** | | | **HER2+ *PIK3CA*m (2nd line)** | | |
| --- | --- | --- | --- | --- | --- | --- | --- | --- | --- |
| **Characteristic** | **aHR** | **95% CI** | **p-value** | **aHR** | **95% CI** | **p-value** | **aHR** | **95% CI** | **p-value** |
| Age at Diagnosis |  |  |  |  |  |  |  |  |  |
| Race |  |  |  |  |  |  |  |  |  |
| White | — | — |  |  |  |  |  |  |  |
| Black | 0.98 | 0.21, 4.66 | >0.9 |  |  |  |  |  |  |
| Asian | 0 | 0.00, Inf | >0.9 |  |  |  |  |  |  |
| Other/Unknown | 0.89 | 0.11, 6.99 | >0.9 |  |  |  |  |  |  |
| Ethnicity |  |  |  |  |  |  |  |  |  |
| Non-Hispanic |  |  |  |  |  |  |  |  |  |
| Hispanic |  |  |  |  |  |  |  |  |  |
| Unknown |  |  |  |  |  |  |  |  |  |
| Tumor Laterality |  |  |  |  |  |  |  |  |  |
| Left |  |  |  |  |  |  |  |  |  |
| Other/Unknown |  |  |  |  |  |  |  |  |  |
| Right |  |  |  |  |  |  |  |  |  |
| Histology |  |  |  |  |  |  |  |  |  |
| Invasive ductal carcinoma |  |  |  |  |  |  |  |  |  |
| Invasive lobular carcinoma |  |  |  |  |  |  |  |  |  |
| Other histology |  |  |  |  |  |  |  |  |  |
| ER Status |  |  |  |  |  |  |  |  |  |
| Positive |  |  |  |  |  |  |  |  |  |
| Negative |  |  |  |  |  |  |  |  |  |
| Borderline |  |  |  |  |  |  |  |  |  |
| Unknown/Not Tested |  |  |  |  |  |  |  |  |  |
| PR Status |  |  |  |  |  |  |  |  |  |
| Positive | — | — |  |  |  |  |  |  |  |
| Negative | 2.32 | 0.75, 7.12 | 0.14 |  |  |  |  |  |  |
| Unknown/Not Tested |  |  |  |  |  |  |  |  |  |
| Breast Cancer Subtype |  |  |  |  |  |  |  |  |  |
| HER2+, HR- |  |  |  |  |  |  |  |  |  |
| HER2+, HR+ |  |  |  |  |  |  |  |  |  |
| Stage at Diagnosis |  |  |  |  |  |  |  |  |  |
| Early Stage (I-III) |  |  |  |  |  |  |  |  |  |
| Stage IV |  |  |  |  |  |  |  |  |  |
| Unknown/Other Stage |  |  |  |  |  |  |  |  |  |
| Received Chemotherapy |  |  |  |  |  |  |  |  |  |
| Yes |  |  |  |  |  |  |  |  |  |
| No |  |  |  |  |  |  |  |  |  |
| Received Hormone Therapy |  |  |  |  |  |  |  |  |  |
| Yes | — | — |  | — | — |  | — | — |  |
| No | 2.78 | 0.87, 8.95 | 0.086 | 4.65 | 1.78, 12.1 | **0.002** | 4.97 | 1.91, 12.9 | **0.001** |
| Received Immunotherapy |  |  |  |  |  |  |  |  |  |
| Yes |  |  |  |  |  |  |  |  |  |
| No |  |  |  |  |  |  |  |  |  |
| Received HER2 Therapy |  |  |  |  |  |  |  |  |  |
| Yes | — | — |  | — | — |  | — | — |  |
| No | 0.38 | 0.09, 1.66 | 0.2 | 0.25 | 0.03, 1.96 | 0.2 | 0.23 | 0.03, 1.80 | 0.2 |
| Received CDK4/6 Inhibitors |  |  |  |  |  |  |  |  |  |
| Yes | — | — |  |  |  |  |  |  |  |
| No | 0.94 | 0.26, 3.39 | >0.9 |  |  |  |  |  |  |
| Received PI3K Inhibitors |  |  |  |  |  |  |  |  |  |
| Yes |  |  |  |  |  |  |  |  |  |
| No |  |  |  |  |  |  |  |  |  |
| Received Other Therapy |  |  |  |  |  |  |  |  |  |
| Yes | — | — |  | — | — |  | — | — |  |
| No | 2.91 | 0.60, 14.2 | 0.2 | 47,358,280 | 0.00, Inf | >0.9 | 44,802,527 | 0.00, Inf | >0.9 |
| TP53 Alteration |  |  |  |  |  |  |  |  |  |
| Altered |  |  |  |  |  |  |  |  |  |
| Not Altered |  |  |  |  |  |  |  |  |  |
| ERBB2 Alteration |  |  |  |  |  |  |  |  |  |
| Altered |  |  |  |  |  |  |  |  |  |
| Not Altered |  |  |  |  |  |  |  |  |  |
| p53 Pathway |  |  |  |  |  |  |  |  |  |
| Altered |  |  |  |  |  |  |  |  |  |
| Not Altered |  |  |  |  |  |  |  |  |  |
| Cell Cycle Pathway |  |  |  |  |  |  |  |  |  |
| Altered |  |  |  |  |  |  |  |  |  |
| Not Altered |  |  |  |  |  |  |  |  |  |

Abbreviations: CI = Confidence Interval, HR = Hazard Ratio, aHR = adjusted Hazard Ratio

**Supplemental Table 18.** Multivariable Cox Proportional Hazard Models for Overall Survival after treatment for HER2+ patients with *PIK3CA* mutation overall and by line of therapy.

|  | **HER2+ *PIK3CA*m** | | | **HER2+ *PIK3CA*m (1st line)** | | | **HER2+ *PIK3CA*m (2nd line)** | | |
| --- | --- | --- | --- | --- | --- | --- | --- | --- | --- |
| **Characteristic** | **aHR** | **95% CI** | **p-value** | **aHR** | **95% CI** | **p-value** | **aHR** | **95% CI** | **p-value** |
| Age at Diagnosis |  |  |  |  |  |  |  |  |  |
| Race |  |  |  |  |  |  |  |  |  |
| White |  |  |  |  |  |  | — | — |  |
| Black |  |  |  |  |  |  | 0.71 | 0.16, 3.20 | 0.7 |
| Asian |  |  |  |  |  |  | 0 | 0.00, Inf | >0.9 |
| Other/Unknown |  |  |  |  |  |  | 1.35 | 0.17, 10.7 | 0.8 |
| Ethnicity |  |  |  |  |  |  |  |  |  |
| Non-Hispanic |  |  |  |  |  |  |  |  |  |
| Hispanic |  |  |  |  |  |  |  |  |  |
| Unknown |  |  |  |  |  |  |  |  |  |
| Tumor Laterality |  |  |  |  |  |  |  |  |  |
| Left |  |  |  |  |  |  |  |  |  |
| Other/Unknown |  |  |  |  |  |  |  |  |  |
| Right |  |  |  |  |  |  |  |  |  |
| Histology |  |  |  |  |  |  |  |  |  |
| Invasive ductal carcinoma |  |  |  |  |  |  |  |  |  |
| Invasive lobular carcinoma |  |  |  |  |  |  |  |  |  |
| Other histology |  |  |  |  |  |  |  |  |  |
| ER Status |  |  |  |  |  |  |  |  |  |
| Positive |  |  |  |  |  |  |  |  |  |
| Negative |  |  |  |  |  |  |  |  |  |
| Borderline |  |  |  |  |  |  |  |  |  |
| Unknown/Not Tested |  |  |  |  |  |  |  |  |  |
| PR Status |  |  |  |  |  |  |  |  |  |
| Positive |  |  |  |  |  |  |  |  |  |
| Negative |  |  |  |  |  |  |  |  |  |
| Unknown/Not Tested |  |  |  |  |  |  |  |  |  |
| Breast Cancer Subtype |  |  |  |  |  |  |  |  |  |
| HER2+, HR- |  |  |  |  |  |  |  |  |  |
| HER2+, HR+ |  |  |  |  |  |  |  |  |  |
| Stage at Diagnosis |  |  |  |  |  |  |  |  |  |
| Early Stage (I-III) |  |  |  |  |  |  |  |  |  |
| Stage IV |  |  |  |  |  |  |  |  |  |
| Unknown/Other Stage |  |  |  |  |  |  |  |  |  |
| Received Chemotherapy |  |  |  |  |  |  |  |  |  |
| Yes |  |  |  |  |  |  |  |  |  |
| No |  |  |  |  |  |  |  |  |  |
| Received Hormone Therapy |  |  |  |  |  |  |  |  |  |
| Yes | — | — |  | — | — |  | — | — |  |
| No | 4.51 | 1.77, 11.5 | **0.002** | 4.67 | 1.79, 12.2 | **0.002** | 5 | 1.89, 13.3 | **0.001** |
| Received Immunotherapy |  |  |  |  |  |  |  |  |  |
| Yes |  |  |  |  |  |  |  |  |  |
| No |  |  |  |  |  |  |  |  |  |
| Received HER2 Therapy |  |  |  |  |  |  |  |  |  |
| Yes | — | — |  | — | — |  | — | — |  |
| No | 0.27 | 0.06, 1.27 | 0.1 | 0.29 | 0.04, 2.28 | 0.2 | 0.26 | 0.03, 2.08 | 0.2 |
| Received CDK4/6 Inhibitors |  |  |  |  |  |  |  |  |  |
| Yes |  |  |  |  |  |  |  |  |  |
| No |  |  |  |  |  |  |  |  |  |
| Received PI3K Inhibitors |  |  |  |  |  |  |  |  |  |
| Yes |  |  |  |  |  |  |  |  |  |
| No |  |  |  |  |  |  |  |  |  |
| Received Other Therapy |  |  |  |  |  |  |  |  |  |
| Yes | — | — |  | — | — |  | — | — |  |
| No | 2.43 | 0.56, 10.4 | 0.2 | 48,414,976 | 0.00, Inf | >0.9 | 59,674,247 | 0.00, Inf | >0.9 |
| TP53 Alteration |  |  |  |  |  |  |  |  |  |
| Altered |  |  |  |  |  |  |  |  |  |
| Not Altered |  |  |  |  |  |  |  |  |  |
| ERBB2 Alteration |  |  |  |  |  |  |  |  |  |
| Altered |  |  |  |  |  |  |  |  |  |
| Not Altered |  |  |  |  |  |  |  |  |  |
| p53 Pathway |  |  |  |  |  |  |  |  |  |
| Altered |  |  |  |  |  |  |  |  |  |
| Not Altered |  |  |  |  |  |  |  |  |  |
| Cell Cycle Pathway |  |  |  |  |  |  |  |  |  |
| Altered |  |  |  |  |  |  |  |  |  |
| Not Altered |  |  |  |  |  |  |  |  |  |

Abbreviations: CI = Confidence Interval, HR = Hazard Ratio, aHR = adjusted Hazard Ratio

**Supplemental Table 19.** Ongoing clinical trials evaluating PI3K/PIK3CA inhibitors in HER2-positive breast cancer.

| **ClinicalTrials.gov ID** | **Title** | **Setting/Stage** | **Agents/Combinations** | **Design** | **Phase** | **Status** | **Estimated Study Completion** |
| --- | --- | --- | --- | --- | --- | --- | --- |
| **NCT05230810** | Clinical Trial of Alpelisb and Tucatinib in Patients With PIK3CA-Mutant HER2+ Metastatic Breast Cancer. | HER2+ locally advanced unresectable or metastatic Breast cancer | Tucatinib + alpelisib ± fulvestrant (HR+ cohort) | Multicenter, Single Arm, Open-label | Phase Ib/II | Active, Not Recruiting | 2026-06 |
| **NCT05063786** | Trastuzumab + Alpelisib +/- Fulvestrant vs Trastuzumab + CT in Patients With PIK3CA Mutated Previously Treated HER2+ Advanced BrEasT Cancer (ALPHABET) | Previously treated HER2+ Advanced Breast cancer | Trastuzumab + alpelisib ± fulvestrant vs trastuzumab + chemotherapy | International, multicenter, open-label, controlled, randomized | Phase III | Active / Not Recruiting | 2026-06 |
| **NCT05306041** | Neoadjuvant Endocrine Therapy +/- the PI3K Inhibitor Inavolisib in HER2+, HR+, PIK3CA Mutant Early Breast Cancer (GeparPiPPa) | Early-stage HER2+, HR-positive | Inavolisib + standard neoadjuvant therapy | Multicenter, prospective, randomized, open-label, parallel-group | Phase II | Recruiting | 2027-01 |
| **NCT04208178** | Study of Alpelisib (BYL719) in Combination With Trastuzumab and Pertuzumab as Maintenance Therapy in Patients With HER2-positive Advanced Breast Cancer With a PIK3CA Mutation (EPIK-B2) | HER2+ Advanced Breast Cancer | Alpelisib + trastuzumab + pertuzumab | Multicenter, randomized, double-blind, placebo-controlled, | Phase III | Active, Not Recruiting | 2027-02 |
| **NCT03424005** | A Study Evaluating the Efficacy and Safety of Multiple Treatment Combinations in Patients With Metastatic or Locally Advanced Breast Cancer (Morpheus-panBC) | Includes a HER2+/HER2-low PIK3CA-mutant cohort after progression on standard HER2 therapy | Multiple combinations including inavolisib + trastuzumab deruxtecan | Open-label, Multicenter, Randomized Umbrella Study | Phase Ib/II | Recruiting | 2030-09 |
| **NCT05894239** | A Study to Evaluate the Efficacy and Safety of Inavolisib in Combination With Phesgo Versus Placebo in Combination With Phesgo in Participants With PIK3CA-Mutated HER2-Positive Locally Advanced or Metastatic Breast Cancer (INAVO122) | HER2+ Locally Advanced or Metastatic Breast Cancer | Inavolisib + Phesgo vs placebo + Phesgo | Multicenter, Randomized, Double-Blind, Placebo-Controlled | Phase III | Recruiting | 2032-12 |

Trials were identified through a search of ClinicalTrials.gov using the condition/disease “breast cancer”, the term “HER2-positive breast cancer,” and interventions/treatments including PIK3CA inhibitor, PI3K inhibitor, alpelisib, inavolisib, buparlisib, taselisib, copanlisib, RLY-2608, or GDC-0077. Trials in the HER2-positive setting, in either early or advanced disease, were included. The search was performed on April 2026.
